# Supplementary material for: Multiscale Modes of Functional Brain Connectivity
Source: bioRxiv. 2024 Jun 1:2024.05.28.596120. Preprint. [Version 1] doi: 10.1101/2024.05.28.596120 (PMC11160636; doi:10.1101/2024.05.28.596120)
Supplement: Supplement 1 [file NIHPP2024.05.28.596120v1-supplement-1.pdf]

bioRxiv preprint doi: https://doi.org/10.1101/2024.05.28.596120; this version posted June 1, 2024. The copyright holder for this preprint (which was not certified by peer review) is the author/funder, who has granted bioRxiv a license to display the preprint in perpetuity. It is made available under aCC-BY 4.0 International license.

7 Supplementary Materials

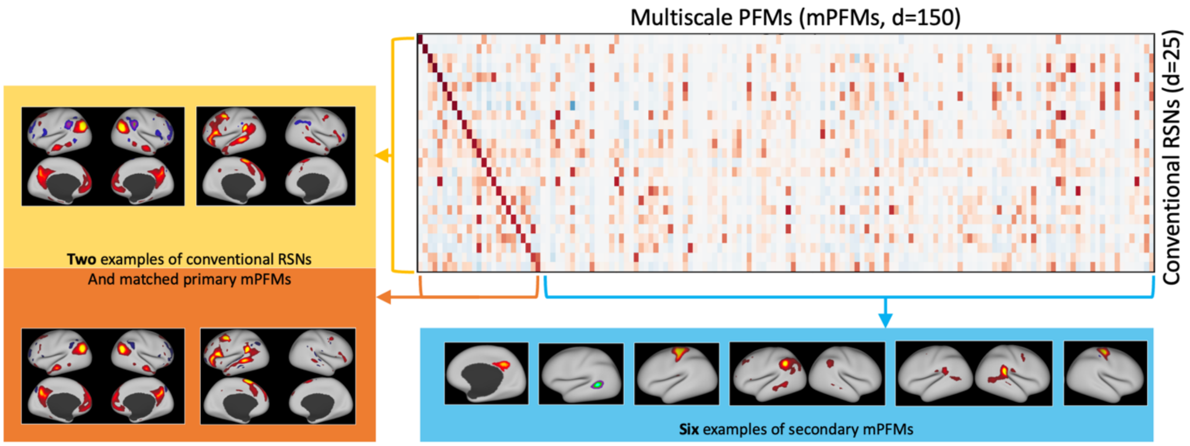

Figure S 1 Supplement to Figure 1: This figure provides an alternative visualisation of the comparison between multiscale Probabilistic Functional Modes (mPFMs) and conventional large-scale RSNs. This visualisation provides additional clarification that when mPFMs are spatially paired with large-scale RSNs from 25-mode decomposition (yellow), 25 of the mPFMs show a clear one-to-one matching, labelled as Primary mPFMs (orange). Secondary mPFMs (blue) are the remaining 125 mPFMs, that start appearing with increased dimensionality.

bioRxiv preprint doi: https://doi.org/10.1101/2024.05.28.596120; this version posted June 1, 2024. The copyright holder for this preprint (which was not certified by peer review) is the author/funder, who has granted bioRxiv a license to display the preprint in perpetuity. It is made available under aCC-BY 4.0 International license.

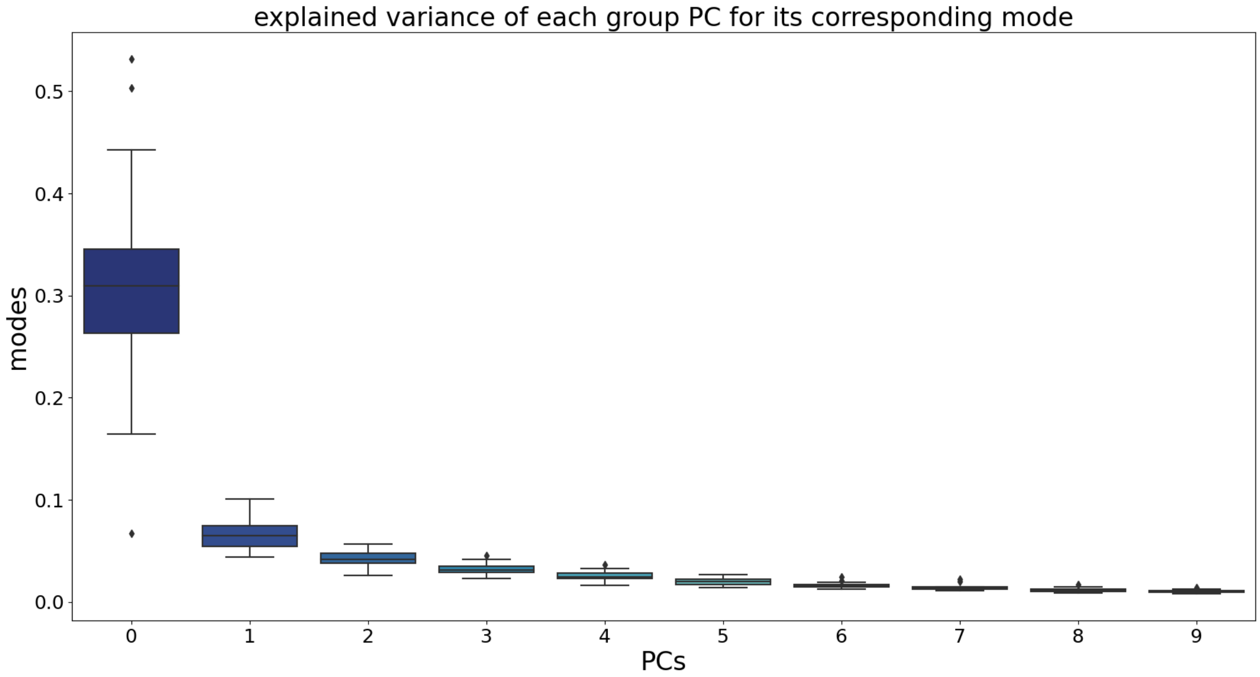

Figure S 2 Supplement to Section "Multiple distinct subcomponents within each conventional RSN": Principal Component Analysis was used to test if one temporal component is sufficient to capture temporal variability within conventional large-scale RSNs from lowD decompositions of rfMRI. LowD refers to 25 modes obtained from PFM decomposition of rfMRI in HCP. The top 10 PCs per lowD mode are illustrated on the x-axis, and their explained variance on the y-axis. Boxplots show median and confidence intervals across 25 modes.

bioRxiv preprint doi: https://doi.org/10.1101/2024.05.28.596120; this version posted June 1, 2024. The copyright holder for this preprint (which was not certified by peer review) is the author/funder, who has granted bioRxiv a license to display the preprint in perpetuity. It is made available under aCC-BY 4.0 International license.

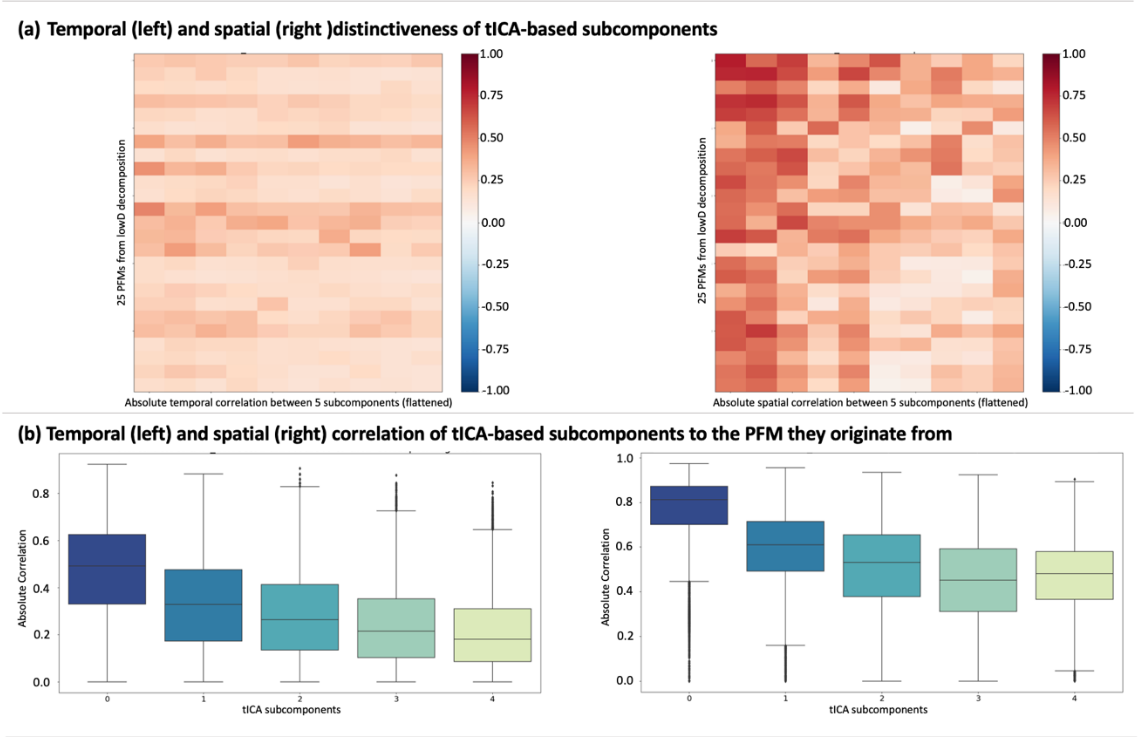

Figure S 3 Supplement to Section "Multiple distinct subcomponents within each conventional RSN": a) The degree to which 5x25 subcomponents estimated using temporal ICA are spatially (right) and temporally (left) correlated with each other (i.e. testing for distinctiveness). The 25 rows correspond to the 25 modes, and the flattened above-diagonal of 5x5 correlation matrices (correlations between subcomponents estimated within-subject and then averaged over subjects) appear on the x-axis. b) Temporal (left) and spatial (right) correlation of 5x25 subcomponents with their respective lowD modes. Boxplots show median and confidence intervals across 25 modes.

bioRxiv preprint doi: https://doi.org/10.1101/2024.05.28.596120; this version posted June 1, 2024. The copyright holder for this preprint (which was not certified by peer review) is the author/funder, who has granted bioRxiv a license to display the preprint in perpetuity. It is made available under aCC-BY 4.0 International license.

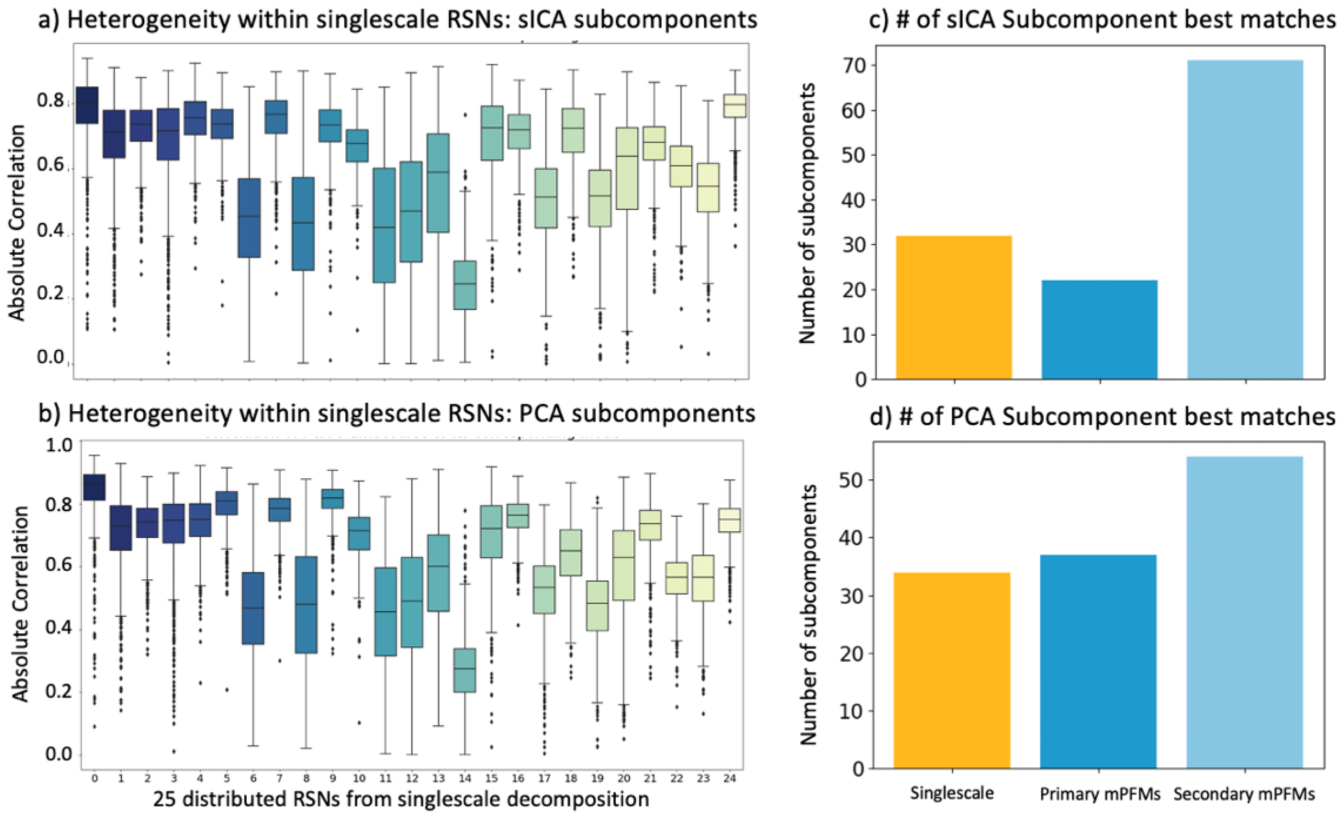

Figure S 4 Supplement to Section "Multiple distinct subcomponents within each conventional RSN": A low-dimensional (lowD) PFM decomposition consisting of 25 modes was estimated from cortical rfMRI data of 1003 HCP subjects, yielding conventional large-scale RSNs, and follow-up spatial ICA (top) and PCA (bottom) applied to voxel-wise timeseries within these RSNs to identify temporally-distinct subcomponents. This analysis was conducted to confirm that subcomponent identification results in section 2.1.1 are not specific to the choice of temporal ICA as the subcomponent identification technique. Temporal correlation of the best-matching a) sICA and b) PCA subcomponents to the large-scale RSNs that they originated from; A winner-takes-all approach was applied: c) of the 25x5 sICA subcomponents 32, 22 and 71 were best represented by large-scale decomposition, primary and secondary mPFMs, respectively; d) of the 25x5 PCA subcomponents 34, 37 and 54 were best represented by large-scale decomposition, primary and secondary mPFMs, respectively.

bioRxiv preprint doi: https://doi.org/10.1101/2024.05.28.596120; this version posted June 1, 2024. The copyright holder for this preprint (which was not certified by peer review) is the author/funder, who has granted bioRxiv a license to display the preprint in perpetuity. It is made available under aCC-BY 4.0 International license.

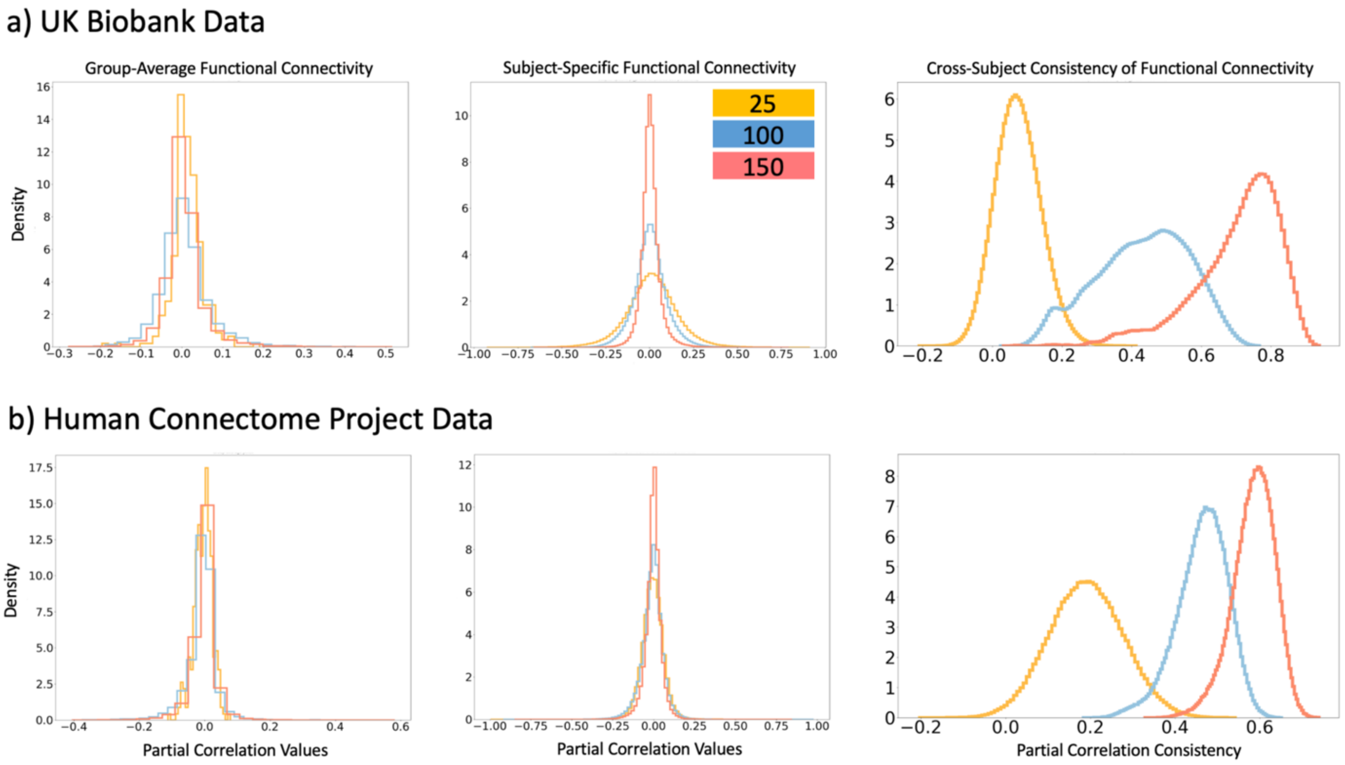

Figure S 5 Supplement to Section "Functional connectivity modelling using mPFMs": Functional connectivity values between modes, estimated using regularised partial correlations. Three PFM dimensionalities are compared: 25, 100 and 150 based on a) 4999 subjects in UK Biobank data (volumetric fMRI) and b) 1003 subjects in Human Connectome Project data (cortical CIFTI). Left: distributions of group average Functional connectivity values. Middle: distributions of subject specific functional connectivity values. Right: Cross subject consistenties of Functional connectivity, which is calculated as Pearson correlation coefficient between vectorised functional connectivity matrices across subjects. Subject-specific functional connectivity becomes sparser and more consistent acorss subjects in mPFMs, leading to less sparse group-average functional connectivity.

bioRxiv preprint doi: https://doi.org/10.1101/2024.05.28.596120; this version posted June 1, 2024. The copyright holder for this preprint (which was not certified by peer review) is the author/funder, who has granted bioRxiv a license to display the preprint in perpetuity. It is made available under aCC-BY 4.0 International license.

28 HCP modes with reproducibility score <0.6 in UKB

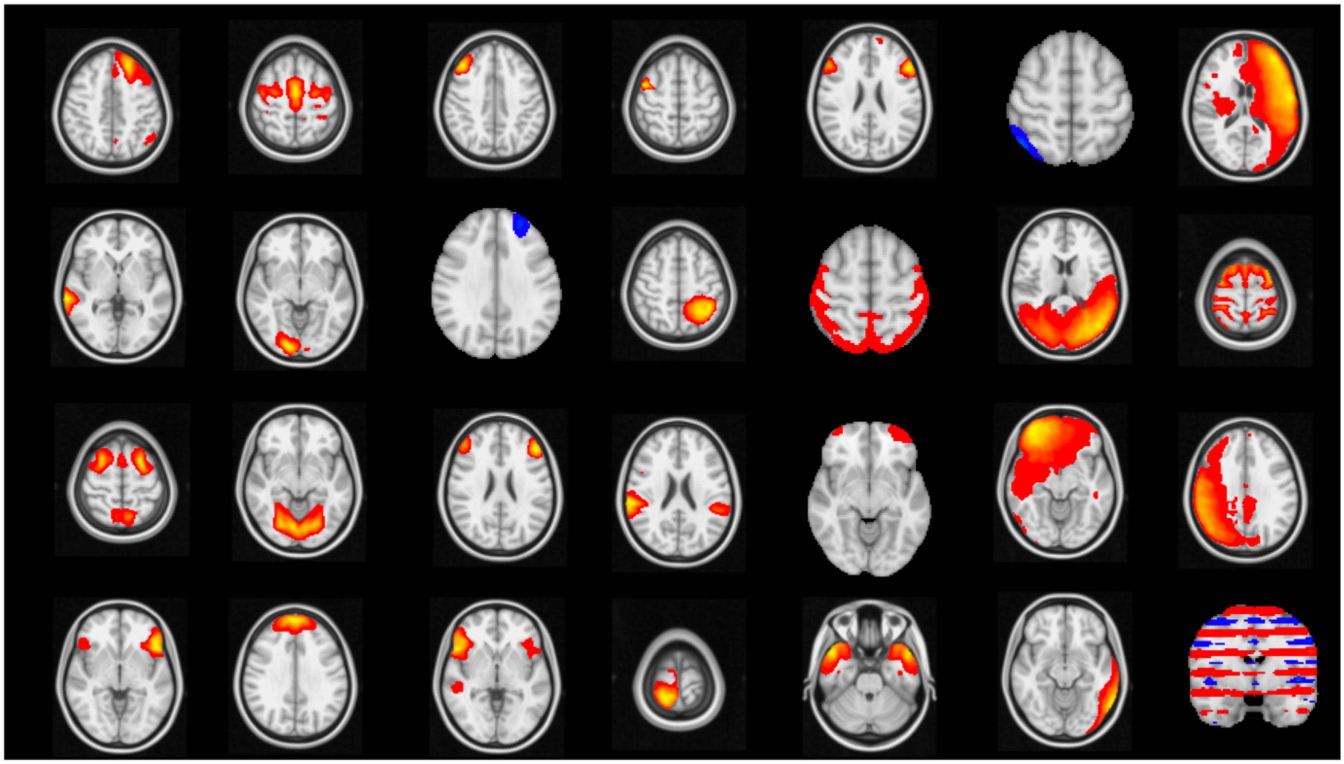

Figure S 6 Supplement to Figure 4: 28 Volumetric HCP modes with reproducibility scores of <0.6 in UKB. Several of these modes were artefactual.

bioRxiv preprint doi: https://doi.org/10.1101/2024.05.28.596120; this version posted June 1, 2024. The copyright holder for this preprint (which was not certified by peer review) is the author/funder, who has granted bioRxiv a license to display the preprint in perpetuity. It is made available under aCC-BY 4.0 International license.

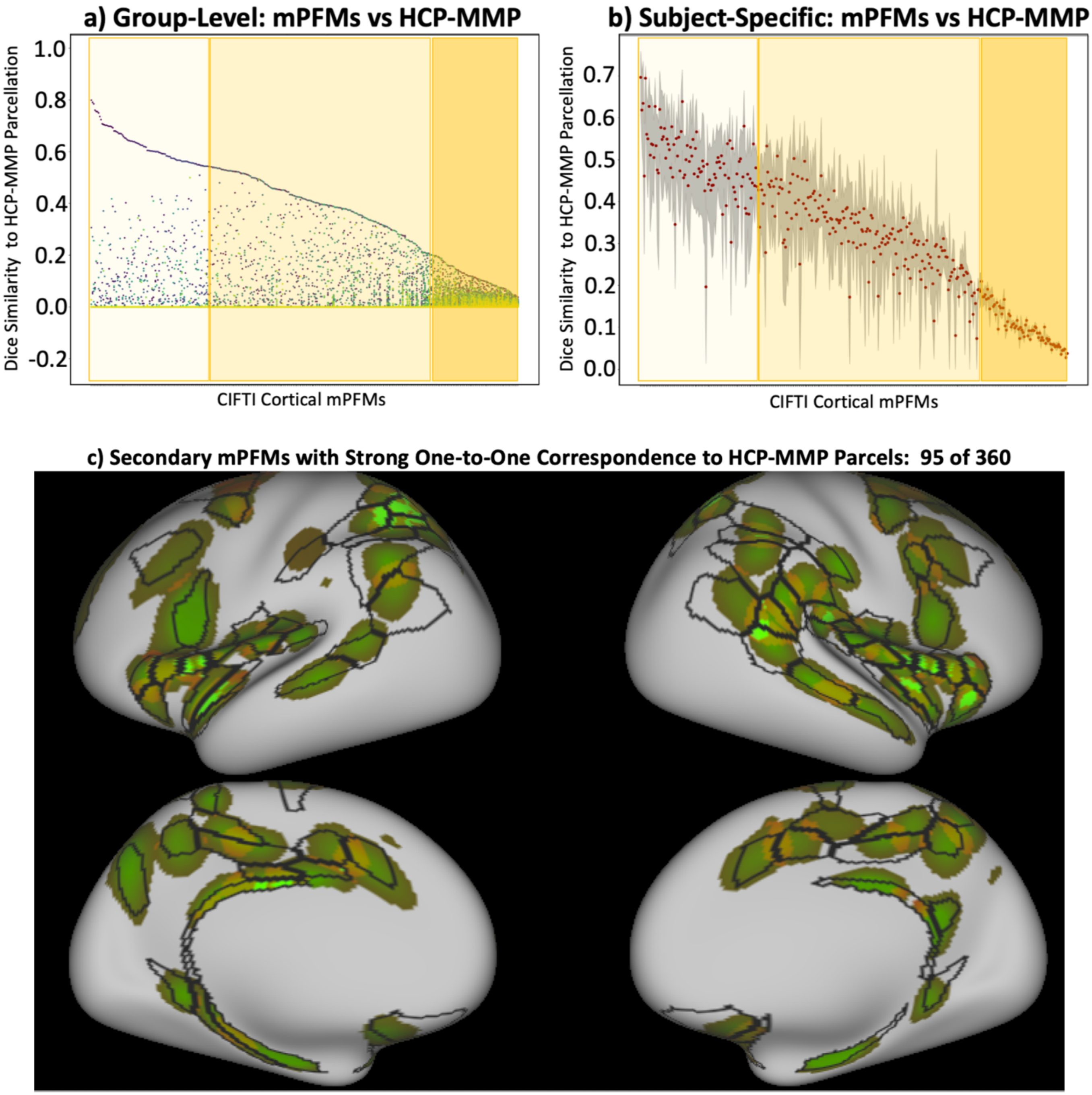

Figure S 7 Supplement to Section "Validating secondary mPFMs using HCP's Multi-Modal Parcellation": a) group-level HCP-MMP parcellation with 360 parcels compared to a 360-mPFM decomposition of HCP. 95 modes were parcel-like (lightest shade of yellow) and showed a clear one-to-one match to HCP-MMP parcels. The remaining large-scale and mixed-scale mPFMs are marked with dark and medium shades of yellow, respectively. 73 mPFMs showed less than 0.2 dice similarity to any HCP-MMP parcels (highlighted with the darkest shade of yellow); these included conventional low-dimensional modes and the less well-known variants of large-scale modes, and typically occupied multiple distant sub-regions. b) subject-level HCP MMP (with the same ordering as Fig5a). Median showed in red, and grey error margins show 25 to 75 percentile across subjects. Good pairing for 95 parcel-like modes were observed here as well. d) Top 95 matched pairs of modes between mPFM and HCP-MMP. Black contours denote group-level HCP-MMP parcels and green-brown patches denote thresholded group-level PFMs.

bioRxiv preprint doi: https://doi.org/10.1101/2024.05.28.596120; this version posted June 1, 2024. The copyright holder for this preprint (which was not certified by peer review) is the author/funder, who has granted bioRxiv a license to display the preprint in perpetuity. It is made available under aCC-BY 4.0 International license.

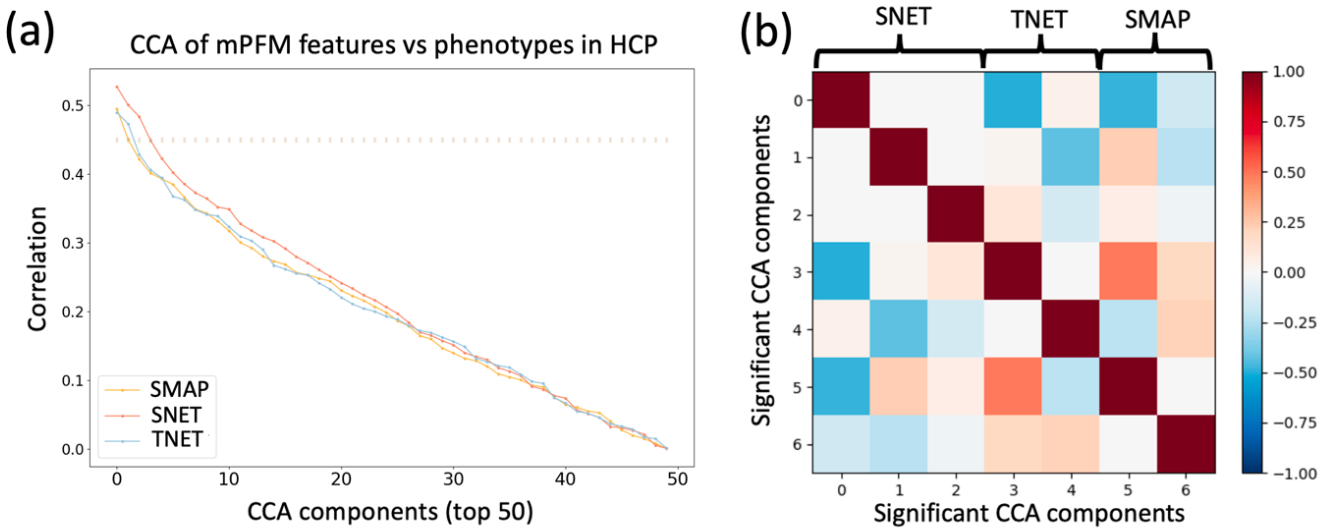

Figure S 8 Supplement to Figure 5: shared population covariation between mPFMs and behaviour in HCP: a) Canonical Correlation Analysis (CCA) was used to compute modes of population co-variation between behavioural traits and spatial maps (SMAPs)/Spatial correlations (SNETs)/temporal correlations (TNETs) of mPFMs. Statistical significance of CCA components was determined using multi-level block permutations that takes family structure of HCP data into account (*54*). b) 2, 3 and 2 significant CCA components were identified for SMAPs, SNETs and TNETs, respectively, and their correlations are shown. we found maximum correlations between transformed pairs SNET-SMAP, SNET-TNET, and SMAP-TNET to be -0.498, -0.484 and 0.483, respectively, indicating that the phenotype-transformed SMAPs, SNETs, and TNETs capture distinct aspects of subject variability.

bioRxiv preprint doi: https://doi.org/10.1101/2024.05.28.596120; this version posted June 1, 2024. The copyright holder for this preprint (which was not certified by peer review) is the author/funder, who has granted bioRxiv a license to display the preprint in perpetuity. It is made available under aCC-BY 4.0 International license.

Table S 1 A summary of terminology used throughout the paper.

Terminology	Description
**Functional Modes**	Modes of functional brain connectivity, where one or multiple brain regions work in synchrony such that they can be described with a single timecourse.
**PFMs**	Probabilistic Functional Modes: functional modes estimated using sPROFUMO software, which estimates the modes simultaneously for big populations and individuals, thus yielding *personalised* functional modes.
**RSNs**	Resting State Networks: functional modes estimated from resting state fMRI (rfMRI). Resting state refers to brain activity from task-free recordings, where participants are instructed to not think about anything specific while lying in the scanner.
**Large-scale or Distributed RSNs/PFMs**	25 modes from low-dimensional (lowD) decomposition of rfMRI data using PFMs.
**Multiscale PFMs (mPFMs)**	Modes from high-dimensional (highD) decomposition of rfMRI data using PFMs. Depending on dataset/application, we have used different number of modes as highD decomposition. Specifically, for HCP data we have used 50, 100, 150 or 360 modes, for UK Biobank data we have used 150 or 100 modes. Justifications are included the corresponding Methods subsections.
**Primary mPFMs**	25 distributed modes from high-dimensional PFM decomposition of rfMRI that best-match the large-scale RSNs.
**Secondary mPFMs**	New modes from high-dimensional PFM decomposition that emerge as we increase the dimensionality to >=100.
**Spatial Maps, or SMAPs**	Spatial organisation of functional modes across the brain voxels.
**Functional Connectivity**	A matrix of size $N_{mode} \times N_{mode}$ including partial temporal correlations between timecourses of functional modes, also referred to as TNET. L2/Tikhonov regularisation is used in estimation of partial correlations.
**Spatial Connectivity**	A matrix of size $N_{mode} \times N_{mode}$ including spatial correlation coefficients between SMAPs of functional modes, also referred to as SNET. This is an indicator of *spatial overlap* between the modes.

bioRxiv preprint doi: https://doi.org/10.1101/2024.05.28.596120; this version posted June 1, 2024. The copyright holder for this preprint (which was not certified by peer review) is the author/funder, who has granted bioRxiv a license to display the preprint in perpetuity. It is made available under aCC-BY 4.0 International license.

Table S 2 T-value and p-values related to subcomponent analysis: original subcomponents. Statistically significant p-values (Bonferroni-corrected threshold to account for multiple comparisons) are shown in bold font. Supplement to **Figure 2**c&d.

Winner: Large-Scale		Winner: Primary mPFMs		Winner: Secondary mPFMs			
t-values	p-values	t-values	p-values	t-values	p-values	t-values	p-values
0.46	6.42e-01	19.81	**5.57e-74**	22.96	**4.70e-94**	23.08	**7.82e-95**
40.45	**7.04e-213**	17.29	**8.47e-59**	14.19	**8.30e-42**	16.34	**2.07e-53**
1.86	6.35e-02	22.53	**2.74e-91**	17.81	**7.82e-62**	29.73	**8.81e-140**
7.60	**6.64e-14**	14.21	**7.29e-42**	4.64	**4.01e-06**	17.66	**5.87e-61**
36.36	**3.06e-185**	10.26	**1.54e-23**	28.59	**6.15e-132**	-0.61	5.43e-01
37.73	**1.35e-194**	5.33	**1.21e-07**	31.04	**9.76e-149**	24.86	**1.12e-106**
15.01	**4.24e-46**	44.60	**3.17e-240**	19.73	**1.70e-73**	15.69	**8.63e-50**
-2.64	8.33e-03	15.12	**1.07e-46**	10.46	**2.31e-24**	10.89	**3.61e-26**
7.04	**3.49e-12**	14.02	**6.60e-41**	24.87	**1.03e-106**	-0.01	9.94e-01
7.98	**4.06e-15**	57.85	**1.13e-321**	10.30	**1.00e-23**	4.77	**2.08e-06**
35.48	**3.05e-179**	23.84	**7.72e-100**	5.45	**6.42e-08**	11.08	**5.24e-27**
15.05	**2.63e-46**	14.47	**3.29e-43**	12.29	**1.95e-32**	40.87	**1.13e-215**
		4.88	**1.26e-06**	4.77	**2.08e-06**	32.10	**5.01e-156**
		16.06	**8.24e-52**	16.35	**1.92e-53**	18.47	**8.46e-66**
		7.10	**2.36e-12**	51.75	**2.76e-285**	26.74	**2.64e-119**
		37.67	**3.76e-194**	9.99	**1.76e-22**	26.53	**7.10e-118**
		10.92	**2.62e-26**	27.84	**8.20e-127**	16.47	**4.19e-54**
		3.23	**1.27e-03**	52.06	**3.32e-287**	35.81	**1.68e-181**
		15.55	**4.96e-49**	17.32	**5.24e-59**	39.36	**1.46e-205**
		17.40	**1.91e-59**	44.44	**3.60e-239**	61.30	**0.00e+00**
		6.98	**5.22e-12**	10.31	**9.63e-24**	29.06	**3.88e-135**
		33.82	**7.52e-168**	31.74	**1.39e-153**	17.77	**1.28e-61**
		37.02	**9.35e-190**	24.91	**5.39e-107**	29.35	**3.66e-137**
		26.00	**2.47e-114**	15.64	**1.69e-49**	11.63	**1.99e-29**
		27.83	**9.80e-127**	24.38	**1.78e-103**	13.61	**7.77e-39**
		16.07	**6.90e-52**	21.47	**2.08e-84**	34.87	**4.61e-175**
		19.13	**9.34e-70**	22.20	**3.81e-89**	9.84	**7.32e-22**
		38.03	**1.39e-196**	38.65	**8.15e-201**	54.03	**3.83e-299**
				61.69	**0.00e+00**	23.29	**2.93e-96**
				27.37	**1.24e-123**	25.14	**1.54e-108**
				27.32	**2.92e-123**	14.85	**2.95e-45**
				49.52	**1.59e-271**	74.34	**0.00e+00**
				88.06	**0.00e+00**	6.00	**2.82e-09**
				44.50	**1.58e-239**	14.35	**1.34e-42**
				3.59	**3.42e-04**	19.80	**6.30e-74**
				11.52	**6.05e-29**	24.71	**1.17e-105**
				9.78	**1.22e-21**	19.73	**1.75e-73**
				22.43	**1.26e-90**	13.38	**1.12e-37**
				11.77	**4.55e-30**	13.04	**5.38e-36**
				40.02	**5.40e-210**	62.63	**0.00e+00**
				34.89	**3.22e-175**	16.04	**1.02e-51**
				14.68	**2.54e-44**	37.97	**3.27e-196**
						49.84	**1.71e-273**

bioRxiv preprint doi: https://doi.org/10.1101/2024.05.28.596120; this version posted June 1, 2024. The copyright holder for this preprint (which was not certified by peer review) is the author/funder, who has granted bioRxiv a license to display the preprint in perpetuity. It is made available under aCC-BY 4.0 International license.

Table S 3 T-value and p-values related to subcomponent analysis: temporally-exclusive subcomponents. Statistically significant p-values (Bonferroni-corrected threshold to account for multiple comparisons) are shown in bold font. Supplement to **Figure 2**e&f.

Winner: Large-Scale				Winner: Primary mPFMs		Winner: Secondary mPFMs	
t-values	p-values	t-values	p-values	t-values	p-values	t-values	p-values
30.98	**2.52e-148**	46.74	**6.02e-254**	1.52	1.28e-01	6.41	**2.20e-10**
28.70	**1.00e-132**	37.99	**2.40e-196**	3.12	**1.88e-03**	15.88	**8.34e-51**
15.48	**1.20e-48**	4.87	**1.31e-06**	4.21	**2.78e-05**	1.55	1.22e-01
4.11	**4.30e-05**	34.01	**3.38e-169**	1.13	2.59e-01	6.07	**1.82e-09**
41.43	**2.13e-219**	20.80	**3.71e-80**			11.45	**1.27e-28**
1.62	1.06e-01	35.79	**2.27e-181**			11.26	**8.68e-28**
15.50	**9.33e-49**	10.34	**7.19e-24**			1.69	9.20e-02
10.65	**3.63e-25**	10.02	**1.32e-22**				
67.89	**0.00e+00**	1.27	2.04e-01				
16.01	**1.50e-51**	62.64	**0.00e+00**				
26.71	**4.26e-119**	37.78	**6.91e-195**				
7.64	**5.18e-14**	37.84	**2.51e-195**				
42.21	**1.46e-224**	25.98	**3.70e-114**				
15.03	**3.57e-46**	34.11	**7.63e-170**				
24.92	**4.92e-107**	42.65	**1.83e-227**				
6.33	**3.61e-10**	10.44	**2.72e-24**				
22.85	**2.21e-93**	22.93	**6.88e-94**				
60.43	**0.00e+00**	58.42	**0.00e+00**				
25.70	**2.91e-112**	25.22	**4.90e-109**				
5.16	**3.02e-07**	26.83	**6.54e-120**				
27.78	**2.27e-126**	22.02	**5.46e-88**				
8.02	**3.00e-15**	8.93	**1.96e-18**				
50.27	**3.33e-276**	54.66	**6.28e-303**				
33.88	**2.63e-168**	3.63	**2.99e-04**				
27.60	**3.55e-125**	3.79	**1.58e-04**				
17.19	**3.05e-58**	2.38	1.75e-02				
13.68	**3.60e-39**	72.20	**0.00e+00**				
71.00	**0.00e+00**	44.52	**1.04e-239**				
43.53	**3.04e-233**	21.80	**1.47e-86**				
34.39	**9.00e-172**	23.88	**3.76e-100**				
30.32	**8.00e-144**	4.15	**3.59e-05**				
35.19	**2.98e-177**	58.48	**0.00e+00**				
38.02	**1.57e-196**	24.23	**1.77e-102**				
33.46	**2.09e-165**	8.54	**4.82e-17**				
28.81	**1.82e-133**	105.56	**0.00e+00**				
23.01	**2.10e-94**	29.69	**1.62e-139**				
21.48	**1.81e-84**	23.61	**2.41e-98**				
49.70	**1.10e-272**	34.32	**2.69e-171**				
30.70	**2.12e-146**	27.49	**2.07e-124**				
20.28	**6.98e-77**	69.98	**0.00e+00**				
16.30	**3.74e-53**	10.83	**6.31e-26**				
24.46	**5.31e-104**	9.19	**2.15e-19**				
63.10	**0.00e+00**	14.30	**2.33e-42**				
3.10	1.97e-03	4.85	**1.40e-06**				
12.88	**3.22e-35**	38.13	**2.64e-197**				
18.38	**3.02e-65**	23.52	**1.00e-97**				
12.50	**2.12e-33**	11.53	**5.92e-29**				
78.92	**0.00e+00**	74.21	**0.00e+00**				
35.71	**8.46e-181**	18.10	**1.39e-63**				
19.95	**7.70e-75**	42.58	**5.12e-227**				
30.69	**2.35e-146**	31.93	**6.42e-155**				
25.75	**1.29e-112**	38.12	**3.17e-197**				
19.17	**4.85e-70**	91.42	**0.00e+00**				
38.45	**1.86e-199**	29.99	**1.49e-141**				
21.55	**5.77e-85**	30.16	**9.74e-143**				
23.77	**2.17e-99**	27.38	**1.14e-123**				
2.54	1.12e-02	22.64	**5.45e-92**				

bioRxiv preprint doi: https://doi.org/10.1101/2024.05.28.596120; this version posted June 1, 2024. The copyright holder for this preprint (which was not certified by peer review) is the author/funder, who has granted bioRxiv a license to display the preprint in perpetuity. It is made available under aCC-BY 4.0 International license.

Table S 4 T-value and p-values related to subcomponent analysis: spatially-exclusive subcomponents. Statistically significant p-values (Bonferroni-corrected threshold to account for multiple comparisons) are shown in bold font. Supplement to **Figure 2**g&h.

Winner: Large-Scale		Winner: Primary mPFMs		Winner: Secondary mPFMs			
t-values	p-values	t-values	p-values	t-values	p-values	t-values	p-values
5.69	**1.66e-08**	6.72	**2.99e-11**	9.19	**2.26e-19**	34.20	**1.78e-170**
5.38	**9.08e-08**	5.23	**2.08e-07**	7.34	**4.28e-13**	26.13	**3.38e-115**
6.52	**1.13e-10**	15.49	**1.18e-48**	26.05	**1.25e-114**	2.76	5.98e-03
13.65	**5.09e-39**	15.99	**2.08e-51**	34.10	**8.66e-170**	40.76	**5.93e-215**
0.13	8.96e-01	21.76	**2.96e-86**	27.08	**1.23e-121**	18.72	**2.89e-67**
26.21	**9.44e-116**	14.93	**1.17e-45**	43.91	**1.10e-235**	23.28	**3.76e-96**
61.72	**0.00e+00**	10.59	**6.59e-25**	5.19	**2.51e-07**	13.26	**4.26e-37**
22.23	**2.76e-89**	18.96	**1.03e-68**	4.75	**2.30e-06**	53.60	**1.43e-296**
16.48	**3.43e-54**	3.97	**7.59e-05**	49.56	**9.49e-272**	57.32	**1.46e-318**
0.59	5.55e-01	38.36	**7.99e-199**	12.44	**3.84e-33**	31.82	**4.05e-154**
5.10	**4.14e-07**	8.41	**1.43e-16**	31.73	**1.77e-153**	9.44	**2.56e-20**
3.46	**5.65e-04**	19.28	**1.02e-70**	3.03	2.49e-03	14.04	**5.04e-41**
3.33	**9.01e-04**	5.47	**5.58e-08**	55.94	**1.62e-310**	34.32	**2.67e-171**
1.65	9.96e-02	52.83	**6.65e-292**	14.90	**1.68e-45**	26.62	**1.73e-118**
8.39	**1.63e-16**	22.88	**1.58e-93**	4.61	**4.54e-06**	49.63	**3.14e-272**
11.01	**1.13e-26**	12.22	**4.24e-32**	22.67	**3.51e-92**	13.06	**4.35e-36**
19.96	**7.23e-75**	3.86	**1.19e-04**	20.11	**7.89e-76**	26.66	**8.40e-119**
21.98	**1.08e-87**	15.40	**3.47e-48**	1.22	2.21e-01	44.86	**7.14e-242**
11.82	**2.91e-30**	4.54	**6.24e-06**	45.25	**1.96e-244**	26.79	**1.24e-119**
15.23	**2.86e-47**	27.83	**9.10e-127**	15.46	**1.67e-48**	21.68	**8.53e-86**
15.47	**1.43e-48**	27.79	**1.69e-126**	15.53	**6.65e-49**	5.08	**4.61e-07**
		17.66	**6.01e-61**	0.24	8.10e-01	31.60	**1.20e-152**
		27.53	**1.12e-124**	22.53	**2.79e-91**	30.37	**3.89e-144**
		15.86	**1.07e-50**	45.45	**1.05e-245**	52.73	**2.57e-291**
		21.31	**2.11e-83**	3.94	**8.72e-05**	14.26	**3.83e-42**
		18.86	**3.69e-68**	36.17	**5.61e-184**	55.50	**7.10e-308**
		23.85	**6.66e-100**	18.62	**1.05e-66**	13.58	**1.05e-38**
				12.19	**5.60e-32**	12.37	**8.18e-33**
				38.93	**1.22e-202**	11.22	**1.35e-27**
				45.34	**5.24e-245**	25.39	**3.53e-110**
				6.91	**8.36e-12**	24.99	**1.66e-107**
				9.67	**3.17e-21**	18.15	**7.23e-64**
				6.76	**2.32e-11**	25.25	**3.02e-109**
				18.88	**2.96e-68**	75.50	**0.00e+00**
				4.75	**2.31e-06**	8.23	**5.80e-16**
				18.66	**6.15e-67**	38.35	**9.55e-199**
				20.49	**3.29e-78**	14.31	**2.02e-42**
				10.18	**3.28e-23**	27.85	**6.72e-127**
						33.14	**3.38e-163**

bioRxiv preprint doi: https://doi.org/10.1101/2024.05.28.596120; this version posted June 1, 2024. The copyright holder for this preprint (which was not certified by peer review) is the author/funder, who has granted bioRxiv a license to display the preprint in perpetuity. It is made available under aCC-BY 4.0 International license.

Table S 5 T-value and Bonferroni-corrected (36 comparisons) p-values to compare phenotype-prediction performance of Spatial and Temporal/Functional connectivity features related to: a) LowD large-scale RSNs vs highD mPFMs and b) within-scale vs cross-scale mPFMs. Pri: Primary, Sec: Secondary, Statistically significant values are shown in bold font. Supplement to **Figure 3**.

	T-Value (corrected P-Value)					
	Spatial connectivity			Temporal connectivity		
	lowD > mPFMs	Pri2Pri > Pri2Sec	Sec2Sec > Pri2Sec	lowD > mPFMs	Pri2Pri > Pri2Sec	Sec2Sec > Pri2Sec
**Cognitive**	-1.464 (0.148)	-1.243 (0.219)	**-3.526 (7.92E-04)**	-0.6182 (0.539)	-1.5678 (0.122)	-0.487 (0.628)
**GMa**	**-21.657 (1.37E-47)**	**-21.017 (3.89E-46)**	**-14.978 (2.21E-31)**	**-4.6115 (8.88E-06)**	**-2.6633 (8.64E-03)**	**3.3587 (1.01E-03)**
**GMt**	**-9.226 (2.81E-16)**	**-12.042 (1.14E-23)**	**-4.916 (2.34E-06)**	-1.0236 (0.31)	-0.1672 (0.867)	**2.0203 (0.045)**
**WM**	**-26.469 (6.31E-94)**	**-25.468 (2.19E-89)**	-0.583 (0.56)	1.0486 (0.295)	**-9.8976 (5.09E-21)**	**7.1892 (2.75E-12)**
**Task-Contrast**	**-15.190 (4.15E-35)**	**-7.165 (1.48E-11)**	**-56.461 (1.94E-124)**	**-7.0706 (2.56E-11)**	-0.6513 (0.516)	**35.8817 (7.73E-89)**
**Blood**	**-7.272 (2.68E-10)**	**-4.296 (5.09E-05)**	0.663 (0.51)	0.2176 (0.828)	**-5.4334 (6.59E-07)**	-0.5626 (0.575)

bioRxiv preprint doi: https://doi.org/10.1101/2024.05.28.596120; this version posted June 1, 2024. The copyright holder for this preprint (which was not certified by peer review) is the author/funder, who has granted bioRxiv a license to display the preprint in perpetuity. It is made available under aCC-BY 4.0 International license.

Table S 6 T-values and Bonferroni-corrected (6 comparisons) p-values to compare phenotype-prediction performance of mPFMs vs spatial ICA (sICA) and Schaefer Parcellation of the same dimensionality (100 modes). Statistically significant values have been shown in bold font. Supplement to **Figure 5**.

	T-value			Corrected P-Value		
	mPFM > sICA	mPFM > Schaefer	sICA > Schaefer	mPFM vs sICA	mPFM vs Schaefer	sICA vs Schaefer
**Cognitive**	1.261	**7.385**	**7.252**	0.212	**3.61E-10**	**6.21E-10**
**GMa**	**15.137**	**26.991**	**23.107**	**8.57E-32**	**7.90E-59**	**8.59E-51**
**GMt**	**13.964**	**33.923**	**31.499**	**9.64E-29**	**2.09E-71**	**3.06E-67**
**WM**	**12.675**	**51.843**	**57.347**	**1.07E-31**	**6.96E-192**	**5.19E-209**
**Task-Contrast**	**14.572**	**14.496**	**5.086**	**1.93E-19**	**2.38E-19**	**5.75E-06**
**Blood**	**7.974**	**17.409**	**17.33**	**1.24E-11**	**3.07E-28**	**4.04E-28**

bioRxiv preprint doi: https://doi.org/10.1101/2024.05.28.596120; this version posted June 1, 2024. The copyright holder for this preprint (which was not certified by peer review) is the author/funder, who has granted bioRxiv a license to display the preprint in perpetuity. It is made available under aCC-BY 4.0 International license.

Table S 7 Names of UK Biobank Imaging Derived Phenotypes (IDPs) related to GM area: 148. Supplement to **Figure 5**.

IDP #	IDP Name	IDP #	IDP Name
1	aparc-a2009s_lh_area_G+S-frontomargin	75	aparc-a2009s_rh_area_G+S-frontomargin
2	aparc-a2009s_lh_area_G+S-occipital-inf	76	aparc-a2009s_rh_area_G+S-occipital-inf
3	aparc-a2009s_lh_area_G+S-paracentral	77	aparc-a2009s_rh_area_G+S-paracentral
4	aparc-a2009s_lh_area_G+S-subcentral	78	aparc-a2009s_rh_area_G+S-subcentral
5	aparc-a2009s_lh_area_G+S-transv-frontopol	79	aparc-a2009s_rh_area_G+S-transv-frontopol
6	aparc-a2009s_lh_area_G+S-cingul-Ant	80	aparc-a2009s_rh_area_G+S-cingul-Ant
7	aparc-a2009s_lh_area_G+S-cingul-Mid-Ant	81	aparc-a2009s_rh_area_G+S-cingul-Mid-Ant
8	aparc-a2009s_lh_area_G+S-cingul-Mid-Post	82	aparc-a2009s_rh_area_G+S-cingul-Mid-Post
9	aparc-a2009s_lh_area_G-cingul-Post-dorsal	83	aparc-a2009s_rh_area_G-cingul-Post-dorsal
10	aparc-a2009s_lh_area_G-cingul-Post-ventral	84	aparc-a2009s_rh_area_G-cingul-Post-ventral
11	aparc-a2009s_lh_area_G-cuneus	85	aparc-a2009s_rh_area_G-cuneus
12	aparc-a2009s_lh_area_G-front-inf-Opercular	86	aparc-a2009s_rh_area_G-front-inf-Opercular
13	aparc-a2009s_lh_area_G-front-inf-Orbital	87	aparc-a2009s_rh_area_G-front-inf-Orbital
14	aparc-a2009s_lh_area_G-front-inf-Triangul	88	aparc-a2009s_rh_area_G-front-inf-Triangul
15	aparc-a2009s_lh_area_G-front-middle	89	aparc-a2009s_rh_area_G-front-middle
16	aparc-a2009s_lh_area_G-front-sup	90	aparc-a2009s_rh_area_G-front-sup
17	aparc-a2009s_lh_area_G-Ins-lg+S-cent-ins	91	aparc-a2009s_rh_area_G-Ins-lg+S-cent-ins
18	aparc-a2009s_lh_area_G-insular-short	92	aparc-a2009s_rh_area_G-insular-short
19	aparc-a2009s_lh_area_G-occipital-middle	93	aparc-a2009s_rh_area_G-occipital-middle
20	aparc-a2009s_lh_area_G-occipital-sup	94	aparc-a2009s_rh_area_G-occipital-sup
21	aparc-a2009s_lh_area_G-oc-temp-lat-fusifor	95	aparc-a2009s_rh_area_G-oc-temp-lat-fusifor
22	aparc-a2009s_lh_area_G-oc-temp-med-Lingual	96	aparc-a2009s_rh_area_G-oc-temp-med-Lingual
23	aparc-a2009s_lh_area_G-oc-temp-med-Parahip	97	aparc-a2009s_rh_area_G-oc-temp-med-Parahip
24	aparc-a2009s_lh_area_G-orbital	98	aparc-a2009s_rh_area_G-orbital
25	aparc-a2009s_lh_area_G-pariet-inf-Angular	99	aparc-a2009s_rh_area_G-pariet-inf-Angular
26	aparc-a2009s_lh_area_G-pariet-inf-Supramar	100	aparc-a2009s_rh_area_G-pariet-inf-Supramar
27	aparc-a2009s_lh_area_G-parietal-sup	101	aparc-a2009s_rh_area_G-parietal-sup
28	aparc-a2009s_lh_area_G-postcentral	102	aparc-a2009s_rh_area_G-postcentral
29	aparc-a2009s_lh_area_G-precentral	103	aparc-a2009s_rh_area_G-precentral
30	aparc-a2009s_lh_area_G-precuneus	104	aparc-a2009s_rh_area_G-precuneus
31	aparc-a2009s_lh_area_G-rectus	105	aparc-a2009s_rh_area_G-rectus
32	aparc-a2009s_lh_area_G-subcallosal	106	aparc-a2009s_rh_area_G-subcallosal
33	aparc-a2009s_lh_area_G-temp-sup-G-T-transv	107	aparc-a2009s_rh_area_G-temp-sup-G-T-transv
34	aparc-a2009s_lh_area_G-temp-sup-Lateral	108	aparc-a2009s_rh_area_G-temp-sup-Lateral
35	aparc-a2009s_lh_area_G-temp-sup-Plan-polar	109	aparc-a2009s_rh_area_G-temp-sup-Plan-polar
36	aparc-a2009s_lh_area_G-temp-sup-Plan-tempo	110	aparc-a2009s_rh_area_G-temp-sup-Plan-tempo
37	aparc-a2009s_lh_area_G-temporal-inf	111	aparc-a2009s_rh_area_G-temporal-inf
38	aparc-a2009s_lh_area_G-temporal-middle	112	aparc-a2009s_rh_area_G-temporal-middle
39	aparc-a2009s_lh_area_Lat-Fis-ant-Horizont	113	aparc-a2009s_rh_area_Lat-Fis-ant-Horizont
40	aparc-a2009s_lh_area_Lat-Fis-ant-Vertical	114	aparc-a2009s_rh_area_Lat-Fis-ant-Vertical
41	aparc-a2009s_lh_area_Lat-Fis-post	115	aparc-a2009s_rh_area_Lat-Fis-post
42	aparc-a2009s_lh_area_Pole-occipital	116	aparc-a2009s_rh_area_Pole-occipital
43	aparc-a2009s_lh_area_Pole-temporal	117	aparc-a2009s_rh_area_Pole-temporal
44	aparc-a2009s_lh_area_S-calcarine	118	aparc-a2009s_rh_area_S-calcarine
45	aparc-a2009s_lh_area_S-central	119	aparc-a2009s_rh_area_S-central
46	aparc-a2009s_lh_area_S-cingul-Marginalis	120	aparc-a2009s_rh_area_S-cingul-Marginalis
47	aparc-a2009s_lh_area_S-circular-insula-ant	121	aparc-a2009s_rh_area_S-circular-insula-ant
48	aparc-a2009s_lh_area_S-circular-insula-inf	122	aparc-a2009s_rh_area_S-circular-insula-inf
49	aparc-a2009s_lh_area_S-circular-insula-sup	123	aparc-a2009s_rh_area_S-circular-insula-sup
50	aparc-a2009s_lh_area_S-collat-transv-ant	124	aparc-a2009s_rh_area_S-collat-transv-ant
51	aparc-a2009s_lh_area_S-collat-transv-post	125	aparc-a2009s_rh_area_S-collat-transv-post
52	aparc-a2009s_lh_area_S-front-inf	126	aparc-a2009s_rh_area_S-front-inf
53	aparc-a2009s_lh_area_S-front-middle	127	aparc-a2009s_rh_area_S-front-middle
54	aparc-a2009s_lh_area_S-front-sup	128	aparc-a2009s_rh_area_S-front-sup
55	aparc-a2009s_lh_area_S-interm-prim-Jensen	129	aparc-a2009s_rh_area_S-interm-prim-Jensen
56	aparc-a2009s_lh_area_S-intrapariet+P-trans	130	aparc-a2009s_rh_area_S-intrapariet+P-trans
57	aparc-a2009s_lh_area_S-oc-middle+Lunatus	131	aparc-a2009s_rh_area_S-oc-middle+Lunatus
58	aparc-a2009s_lh_area_S-oc-sup+transversal	132	aparc-a2009s_rh_area_S-oc-sup+transversal
59	aparc-a2009s_lh_area_S-occipital-ant	133	aparc-a2009s_rh_area_S-occipital-ant
60	aparc-a2009s_lh_area_S-oc-temp-lat	134	aparc-a2009s_rh_area_S-oc-temp-lat
61	aparc-a2009s_lh_area_S-oc-temp-med+Lingual	135	aparc-a2009s_rh_area_S-oc-temp-med+Lingual
62	aparc-a2009s_lh_area_S-orbital-lateral	136	aparc-a2009s_rh_area_S-orbital-lateral
63	aparc-a2009s_lh_area_S-orbital-med-olfact	137	aparc-a2009s_rh_area_S-orbital-med-olfact
64	aparc-a2009s_lh_area_S-orbital-H-Shaped	138	aparc-a2009s_rh_area_S-orbital-H-Shaped
65	aparc-a2009s_lh_area_S-parieto-occipital	139	aparc-a2009s_rh_area_S-parieto-occipital
66	aparc-a2009s_lh_area_S-pericallosal	140	aparc-a2009s_rh_area_S-pericallosal
67	aparc-a2009s_lh_area_S-postcentral	141	aparc-a2009s_rh_area_S-postcentral
68	aparc-a2009s_lh_area_S-precentral-inf-part	142	aparc-a2009s_rh_area_S-precentral-inf-part
69	aparc-a2009s_lh_area_S-precentral-sup-part	143	aparc-a2009s_rh_area_S-precentral-sup-part
70	aparc-a2009s_lh_area_S-suborbital	144	aparc-a2009s_rh_area_S-suborbital
71	aparc-a2009s_lh_area_S-subparietal	145	aparc-a2009s_rh_area_S-subparietal
72	aparc-a2009s_lh_area_S-temporal-inf	146	aparc-a2009s_rh_area_S-temporal-inf
73	aparc-a2009s_lh_area_S-temporal-sup	147	aparc-a2009s_rh_area_S-temporal-sup
74	aparc-a2009s_lh_area_S-temporal-transverse	148	aparc-a2009s_rh_area_S-temporal-transverse

bioRxiv preprint doi: https://doi.org/10.1101/2024.05.28.596120; this version posted June 1, 2024. The copyright holder for this preprint (which was not certified by peer review) is the author/funder, who has granted bioRxiv a license to display the preprint in perpetuity. It is made available under aCC-BY 4.0 International license.

Table S 8 Names of UK Biobank Imaging Derived Phenotypes (IDPs) related to Grey Matter thickness: 148. Supplement to **Figure 5**.

IDP #	IDP Name	IDP #	IDP Name
1	aparc-a2009s_lh_thickness_G+S-frontomargin,	75	aparc-a2009s_rh_thickness_G+S-frontomargin,
2	aparc-a2009s_lh_thickness_G+S-occipital-inf,	76	aparc-a2009s_rh_thickness_G+S-occipital-inf,
3	aparc-a2009s_lh_thickness_G+S-paracentral,	77	aparc-a2009s_rh_thickness_G+S-paracentral,
4	aparc-a2009s_lh_thickness_G+S-subcentral,	78	aparc-a2009s_rh_thickness_G+S-subcentral,
5	aparc-a2009s_lh_thickness_G+S-transv-frontopol,	79	aparc-a2009s_rh_thickness_G+S-transv-frontopol,
6	aparc-a2009s_lh_thickness_G+S-cingul-Ant,	80	aparc-a2009s_rh_thickness_G+S-cingul-Ant,
7	aparc-a2009s_lh_thickness_G+S-cingul-Mid-Ant,	81	aparc-a2009s_rh_thickness_G+S-cingul-Mid-Ant,
8	aparc-a2009s_lh_thickness_G+S-cingul-Mid-Post,	82	aparc-a2009s_rh_thickness_G+S-cingul-Mid-Post,
9	aparc-a2009s_lh_thickness_G-cingul-Post-dorsal,	83	aparc-a2009s_rh_thickness_G-cingul-Post-dorsal,
10	aparc-a2009s_lh_thickness_G-cingul-Post-ventral,	84	aparc-a2009s_rh_thickness_G-cingul-Post-ventral,
11	aparc-a2009s_lh_thickness_G-cuneus,	85	aparc-a2009s_rh_thickness_G-cuneus,
12	aparc-a2009s_lh_thickness_G-front-inf-Opercular,	86	aparc-a2009s_rh_thickness_G-front-inf-Opercular,
13	aparc-a2009s_lh_thickness_G-front-inf-Orbital,	87	aparc-a2009s_rh_thickness_G-front-inf-Orbital,
14	aparc-a2009s_lh_thickness_G-front-inf-Triangul,	88	aparc-a2009s_rh_thickness_G-front-inf-Triangul,
15	aparc-a2009s_lh_thickness_G-front-middle,	89	aparc-a2009s_rh_thickness_G-front-middle,
16	aparc-a2009s_lh_thickness_G-front-sup,	90	aparc-a2009s_rh_thickness_G-front-sup,
17	aparc-a2009s_lh_thickness_G-Ins-lg+S-cent-ins,	91	aparc-a2009s_rh_thickness_G-Ins-lg+S-cent-ins,
18	aparc-a2009s_lh_thickness_G-insular-short,	92	aparc-a2009s_rh_thickness_G-insular-short,
19	aparc-a2009s_lh_thickness_G-occipital-middle,	93	aparc-a2009s_rh_thickness_G-occipital-middle,
20	aparc-a2009s_lh_thickness_G-occipital-sup,	94	aparc-a2009s_rh_thickness_G-occipital-sup,
21	aparc-a2009s_lh_thickness_G-oc-temp-lat-fusifor,	95	aparc-a2009s_rh_thickness_G-oc-temp-lat-fusifor,
22	aparc-a2009s_lh_thickness_G-oc-temp-med-Lingual,	96	aparc-a2009s_rh_thickness_G-oc-temp-med-Lingual,
23	aparc-a2009s_lh_thickness_G-oc-temp-med-Parahip,	97	aparc-a2009s_rh_thickness_G-oc-temp-med-Parahip,
24	aparc-a2009s_lh_thickness_G-orbital,	98	aparc-a2009s_rh_thickness_G-orbital,
25	aparc-a2009s_lh_thickness_G-pariet-inf-Angular,	99	aparc-a2009s_rh_thickness_G-pariet-inf-Angular,
26	aparc-a2009s_lh_thickness_G-pariet-inf-Supramar,	100	aparc-a2009s_rh_thickness_G-pariet-inf-Supramar,
27	aparc-a2009s_lh_thickness_G-parietal-sup,	101	aparc-a2009s_rh_thickness_G-parietal-sup,
28	aparc-a2009s_lh_thickness_G-postcentral,	102	aparc-a2009s_rh_thickness_G-postcentral,
29	aparc-a2009s_lh_thickness_G-precentral,	103	aparc-a2009s_rh_thickness_G-precentral,
30	aparc-a2009s_lh_thickness_G-precuneus,	104	aparc-a2009s_rh_thickness_G-precuneus,
31	aparc-a2009s_lh_thickness_G-rectus,	105	aparc-a2009s_rh_thickness_G-rectus,
32	aparc-a2009s_lh_thickness_G-subcallosal,	106	aparc-a2009s_rh_thickness_G-subcallosal,
33	aparc-a2009s_lh_thickness_G-temp-sup-G-T-transv,	107	aparc-a2009s_rh_thickness_G-temp-sup-G-T-transv,
34	aparc-a2009s_lh_thickness_G-temp-sup-Lateral,	108	aparc-a2009s_rh_thickness_G-temp-sup-Lateral,
35	aparc-a2009s_lh_thickness_G-temp-sup-Plan-polar,	109	aparc-a2009s_rh_thickness_G-temp-sup-Plan-polar,
36	aparc-a2009s_lh_thickness_G-temp-sup-Plan-tempo,	110	aparc-a2009s_rh_thickness_G-temp-sup-Plan-tempo,
37	aparc-a2009s_lh_thickness_G-temporal-inf,	111	aparc-a2009s_rh_thickness_G-temporal-inf,
38	aparc-a2009s_lh_thickness_G-temporal-middle,	112	aparc-a2009s_rh_thickness_G-temporal-middle,
39	aparc-a2009s_lh_thickness_Lat-Fis-ant-Horizont,	113	aparc-a2009s_rh_thickness_Lat-Fis-ant-Horizont,
40	aparc-a2009s_lh_thickness_Lat-Fis-ant-Vertical,	114	aparc-a2009s_rh_thickness_Lat-Fis-ant-Vertical,
41	aparc-a2009s_lh_thickness_Lat-Fis-post,	115	aparc-a2009s_rh_thickness_Lat-Fis-post,
42	aparc-a2009s_lh_thickness_Pole-occipital,	116	aparc-a2009s_rh_thickness_Pole-occipital,
43	aparc-a2009s_lh_thickness_Pole-temporal,	117	aparc-a2009s_rh_thickness_Pole-temporal,
44	aparc-a2009s_lh_thickness_S-calcarine,	118	aparc-a2009s_rh_thickness_S-calcarine,
45	aparc-a2009s_lh_thickness_S-central,	119	aparc-a2009s_rh_thickness_S-central,
46	aparc-a2009s_lh_thickness_S-cingul-Marginalis,	120	aparc-a2009s_rh_thickness_S-cingul-Marginalis,
47	aparc-a2009s_lh_thickness_S-circular-insula-ant,	121	aparc-a2009s_rh_thickness_S-circular-insula-ant,
48	aparc-a2009s_lh_thickness_S-circular-insula-inf,	122	aparc-a2009s_rh_thickness_S-circular-insula-inf,
49	aparc-a2009s_lh_thickness_S-circular-insula-sup,	123	aparc-a2009s_rh_thickness_S-circular-insula-sup,
50	aparc-a2009s_lh_thickness_S-collat-transv-ant,	124	aparc-a2009s_rh_thickness_S-collat-transv-ant,
51	aparc-a2009s_lh_thickness_S-collat-transv-post,	125	aparc-a2009s_rh_thickness_S-collat-transv-post,
52	aparc-a2009s_lh_thickness_S-front-inf,	126	aparc-a2009s_rh_thickness_S-front-inf,
53	aparc-a2009s_lh_thickness_S-front-middle,	127	aparc-a2009s_rh_thickness_S-front-middle,
54	aparc-a2009s_lh_thickness_S-front-sup,	128	aparc-a2009s_rh_thickness_S-front-sup,
55	aparc-a2009s_lh_thickness_S-interm-prim-Jensen,	129	aparc-a2009s_rh_thickness_S-interm-prim-Jensen,
56	aparc-a2009s_lh_thickness_S-intrapariet+P-trans,	130	aparc-a2009s_rh_thickness_S-intrapariet+P-trans,
57	aparc-a2009s_lh_thickness_S-oc-middle+Lunatus,	131	aparc-a2009s_rh_thickness_S-oc-middle+Lunatus,
58	aparc-a2009s_lh_thickness_S-oc-sup+transversal,	132	aparc-a2009s_rh_thickness_S-oc-sup+transversal,
59	aparc-a2009s_lh_thickness_S-occipital-ant,	133	aparc-a2009s_rh_thickness_S-occipital-ant,
60	aparc-a2009s_lh_thickness_S-oc-temp-lat,	134	aparc-a2009s_rh_thickness_S-oc-temp-lat,
61	aparc-a2009s_lh_thickness_S-oc-temp-med+Lingual,	135	aparc-a2009s_rh_thickness_S-oc-temp-med+Lingual,
62	aparc-a2009s_lh_thickness_S-orbital-lateral,	136	aparc-a2009s_rh_thickness_S-orbital-lateral,
63	aparc-a2009s_lh_thickness_S-orbital-med-olfact,	137	aparc-a2009s_rh_thickness_S-orbital-med-olfact,
64	aparc-a2009s_lh_thickness_S-orbital-H-Shaped,	138	aparc-a2009s_rh_thickness_S-orbital-H-Shaped,
65	aparc-a2009s_lh_thickness_S-parieto-occipital,	139	aparc-a2009s_rh_thickness_S-parieto-occipital,
66	aparc-a2009s_lh_thickness_S-pericallosal,	140	aparc-a2009s_rh_thickness_S-pericallosal,
67	aparc-a2009s_lh_thickness_S-postcentral,	141	aparc-a2009s_rh_thickness_S-postcentral,
68	aparc-a2009s_lh_thickness_S-precentral-inf-part,	142	aparc-a2009s_rh_thickness_S-precentral-inf-part,
69	aparc-a2009s_lh_thickness_S-precentral-sup-part,	143	aparc-a2009s_rh_thickness_S-precentral-sup-part,
70	aparc-a2009s_lh_thickness_S-suborbital,	144	aparc-a2009s_rh_thickness_S-suborbital,
71	aparc-a2009s_lh_thickness_S-subparietal,	145	aparc-a2009s_rh_thickness_S-subparietal,
72	aparc-a2009s_lh_thickness_S-temporal-inf,	146	aparc-a2009s_rh_thickness_S-temporal-inf,
73	aparc-a2009s_lh_thickness_S-temporal-sup,	147	aparc-a2009s_rh_thickness_S-temporal-sup,
74	aparc-a2009s_lh_thickness_S-temporal-transverse,	148	aparc-a2009s_rh_thickness_S-temporal-transverse,

bioRxiv preprint doi: https://doi.org/10.1101/2024.05.28.596120; this version posted June 1, 2024. The copyright holder for this preprint (which was not certified by peer review) is the author/funder, who has granted bioRxiv a license to display the preprint in perpetuity. It is made available under aCC-BY 4.0 International license.

Table S 9 Names of UK Biobank Imaging Derived Phenotypes (IDPs) related to White Matter: 453. Supplement to **Figure 5**.

IDP #	IDP Name	IDP #	IDP Name
1	IDP_T2_FLAIR_BIANCA_WMH_volume	78	IDP_dMRI_ProbtrackX_FA_unc_r
2	IDP_T2_FLAIR_BIANCA_periventWMH_volume	79	IDP_dMRI_TBSS_MO_Middle_cerebellar_peduncle
3	IDP_T2_FLAIR_BIANCA_deepWMH_volume	80	IDP_dMRI_TBSS_MO_Pontine_crossing_tract
4	IDP_dMRI_TBSS_FA_Middle_cerebellar_peduncle	81	IDP_dMRI_TBSS_MO_Genu_of_corpus_callosum
5	IDP_dMRI_TBSS_FA_Pontine_crossing_tract	82	IDP_dMRI_TBSS_MO_Body_of_corpus_callosum
6	IDP_dMRI_TBSS_FA_Genu_of_corpus_callosum	83	IDP_dMRI_TBSS_MO_Splenium_of_corpus_callosum
7	IDP_dMRI_TBSS_FA_Body_of_corpus_callosum	84	IDP_dMRI_TBSS_MO_Fornix
8	IDP_dMRI_TBSS_FA_Splenium_of_corpus_callosum	85	IDP_dMRI_TBSS_MO_Corticospinal_tract_R
9	IDP_dMRI_TBSS_FA_Fornix	86	IDP_dMRI_TBSS_MO_Corticospinal_tract_L
10	IDP_dMRI_TBSS_FA_Corticospinal_tract_R	87	IDP_dMRI_TBSS_MO_Medial_lemniscus_R
11	IDP_dMRI_TBSS_FA_Corticospinal_tract_L	88	IDP_dMRI_TBSS_MO_Medial_lemniscus_L
12	IDP_dMRI_TBSS_FA_Medial_lemniscus_R	89	IDP_dMRI_TBSS_MO_Inferior_cerebellar_peduncle_R
13	IDP_dMRI_TBSS_FA_Medial_lemniscus_L	90	IDP_dMRI_TBSS_MO_Inferior_cerebellar_peduncle_L
14	IDP_dMRI_TBSS_FA_Inferior_cerebellar_peduncle_R	91	IDP_dMRI_TBSS_MO_Superior_cerebellar_peduncle_R
15	IDP_dMRI_TBSS_FA_Inferior_cerebellar_peduncle_L	92	IDP_dMRI_TBSS_MO_Superior_cerebellar_peduncle_L
16	IDP_dMRI_TBSS_FA_Superior_cerebellar_peduncle_R	93	IDP_dMRI_TBSS_MO_Cerebral_peduncle_R
17	IDP_dMRI_TBSS_FA_Superior_cerebellar_peduncle_L	94	IDP_dMRI_TBSS_MO_Cerebral_peduncle_L
18	IDP_dMRI_TBSS_FA_Cerebral_peduncle_R	95	IDP_dMRI_TBSS_MO_Anterior_limb_of_internal_capsule_R
19	IDP_dMRI_TBSS_FA_Cerebral_peduncle_L	96	IDP_dMRI_TBSS_MO_Anterior_limb_of_internal_capsule_L
20	IDP_dMRI_TBSS_FA_Anterior_limb_of_internal_capsule_R	97	IDP_dMRI_TBSS_MO_Posterior_limb_of_internal_capsule_R
21	IDP_dMRI_TBSS_FA_Anterior_limb_of_internal_capsule_L	98	IDP_dMRI_TBSS_MO_Posterior_limb_of_internal_capsule_L
22	IDP_dMRI_TBSS_FA_Posterior_limb_of_internal_capsule_R	99	IDP_dMRI_TBSS_MO_Retrolenticular_part_of_internal_capsule_R
23	IDP_dMRI_TBSS_FA_Posterior_limb_of_internal_capsule_L	100	IDP_dMRI_TBSS_MO_Retrolenticular_part_of_internal_capsule_L
24	IDP_dMRI_TBSS_FA_Retrolenticular_part_of_internal_capsule_R	101	IDP_dMRI_TBSS_MO_Anterior_corona_radiata_R
25	IDP_dMRI_TBSS_FA_Retrolenticular_part_of_internal_capsule_L	102	IDP_dMRI_TBSS_MO_Anterior_corona_radiata_L
26	IDP_dMRI_TBSS_FA_Anterior_corona_radiata_R	103	IDP_dMRI_TBSS_MO_Superior_corona_radiata_R
27	IDP_dMRI_TBSS_FA_Anterior_corona_radiata_L	104	IDP_dMRI_TBSS_MO_Superior_corona_radiata_L
28	IDP_dMRI_TBSS_FA_Superior_corona_radiata_R	105	IDP_dMRI_TBSS_MO_Posterior_corona_radiata_R
29	IDP_dMRI_TBSS_FA_Superior_corona_radiata_L	106	IDP_dMRI_TBSS_MO_Posterior_corona_radiata_L
30	IDP_dMRI_TBSS_FA_Posterior_corona_radiata_R	107	IDP_dMRI_TBSS_MO_Posterior_thalamic_radiation_R
31	IDP_dMRI_TBSS_FA_Posterior_corona_radiata_L	108	IDP_dMRI_TBSS_MO_Posterior_thalamic_radiation_L
32	IDP_dMRI_TBSS_FA_Posterior_thalamic_radiation_R	109	IDP_dMRI_TBSS_MO_Sagittal_stratum_R
33	IDP_dMRI_TBSS_FA_Posterior_thalamic_radiation_L	110	IDP_dMRI_TBSS_MO_Sagittal_stratum_L
34	IDP_dMRI_TBSS_FA_Sagittal_stratum_R	111	IDP_dMRI_TBSS_MO_External_capsule_R
35	IDP_dMRI_TBSS_FA_Sagittal_stratum_L	112	IDP_dMRI_TBSS_MO_External_capsule_L
36	IDP_dMRI_TBSS_FA_External_capsule_R	113	IDP_dMRI_TBSS_MO_Cingulum_cingulate_gyrus_R
37	IDP_dMRI_TBSS_FA_External_capsule_L	114	IDP_dMRI_TBSS_MO_Cingulum_cingulate_gyrus_L
38	IDP_dMRI_TBSS_FA_Cingulum_cingulate_gyrus_R	115	IDP_dMRI_TBSS_MO_Cingulum_hippocampus_R
39	IDP_dMRI_TBSS_FA_Cingulum_cingulate_gyrus_L	116	IDP_dMRI_TBSS_MO_Cingulum_hippocampus_L
40	IDP_dMRI_TBSS_FA_Cingulum_hippocampus_R	117	IDP_dMRI_TBSS_MO_Fornix_cres+Stria_terminalis_R
41	IDP_dMRI_TBSS_FA_Cingulum_hippocampus_L	118	IDP_dMRI_TBSS_MO_Fornix_cres+Stria_terminalis_L
42	IDP_dMRI_TBSS_FA_Fornix_cres+Stria_terminalis_R	119	IDP_dMRI_TBSS_MO_Superior_longitudinal_fasciculus_R
43	IDP_dMRI_TBSS_FA_Fornix_cres+Stria_terminalis_L	120	IDP_dMRI_TBSS_MO_Superior_longitudinal_fasciculus_L
44	IDP_dMRI_TBSS_FA_Superior_longitudinal_fasciculus_R	121	IDP_dMRI_TBSS_MO_Superior_fronto-occipital_fasciculus_R
45	IDP_dMRI_TBSS_FA_Superior_longitudinal_fasciculus_L	122	IDP_dMRI_TBSS_MO_Superior_fronto-occipital_fasciculus_L
46	IDP_dMRI_TBSS_FA_Superior_fronto-occipital_fasciculus_R	123	IDP_dMRI_TBSS_MO_Uncinate_fasciculus_R
47	IDP_dMRI_TBSS_FA_Superior_fronto-occipital_fasciculus_L	124	IDP_dMRI_TBSS_MO_Uncinate_fasciculus_L
48	IDP_dMRI_TBSS_FA_Uncinate_fasciculus_R	125	IDP_dMRI_TBSS_MO_Tapetum_R
49	IDP_dMRI_TBSS_FA_Uncinate_fasciculus_L	126	IDP_dMRI_TBSS_MO_Tapetum_L
50	IDP_dMRI_TBSS_FA_Tapetum_R	127	IDP_dMRI_ProbtrackX_MO_ar_l
51	IDP_dMRI_TBSS_FA_Tapetum_L	128	IDP_dMRI_ProbtrackX_MO_ar_r
52	IDP_dMRI_ProbtrackX_FA_ar_l	129	IDP_dMRI_ProbtrackX_MO_atr_l
53	IDP_dMRI_ProbtrackX_FA_ar_r	130	IDP_dMRI_ProbtrackX_MO_atr_r
54	IDP_dMRI_ProbtrackX_FA_atr_l	131	IDP_dMRI_ProbtrackX_MO_cgc_l
55	IDP_dMRI_ProbtrackX_FA_atr_r	132	IDP_dMRI_ProbtrackX_MO_cgc_r
56	IDP_dMRI_ProbtrackX_FA_cgc_l	133	IDP_dMRI_ProbtrackX_MO_cgh_l
57	IDP_dMRI_ProbtrackX_FA_cgc_r	134	IDP_dMRI_ProbtrackX_MO_cgh_r
58	IDP_dMRI_ProbtrackX_FA_cgh_l	135	IDP_dMRI_ProbtrackX_MO_cst_l
59	IDP_dMRI_ProbtrackX_FA_cgh_r	136	IDP_dMRI_ProbtrackX_MO_cst_r
60	IDP_dMRI_ProbtrackX_FA_cst_l	137	IDP_dMRI_ProbtrackX_MO_fma
61	IDP_dMRI_ProbtrackX_FA_cst_r	138	IDP_dMRI_ProbtrackX_MO_fmi
62	IDP_dMRI_ProbtrackX_FA_fma	139	IDP_dMRI_ProbtrackX_MO_ifo_l
63	IDP_dMRI_ProbtrackX_FA_fmi	140	IDP_dMRI_ProbtrackX_MO_ifo_r
64	IDP_dMRI_ProbtrackX_FA_ifo_l	141	IDP_dMRI_ProbtrackX_MO_ilf_l
65	IDP_dMRI_ProbtrackX_FA_ifo_r	142	IDP_dMRI_ProbtrackX_MO_ilf_r
66	IDP_dMRI_ProbtrackX_FA_ilf_l	143	IDP_dMRI_ProbtrackX_MO_mcp
67	IDP_dMRI_ProbtrackX_FA_ilf_r	144	IDP_dMRI_ProbtrackX_MO_ml_l
68	IDP_dMRI_ProbtrackX_FA_mcp	145	IDP_dMRI_ProbtrackX_MO_ml_r
69	IDP_dMRI_ProbtrackX_FA_ml_l	146	IDP_dMRI_ProbtrackX_MO_ptr_l
70	IDP_dMRI_ProbtrackX_FA_ml_r	147	IDP_dMRI_ProbtrackX_MO_ptr_r
71	IDP_dMRI_ProbtrackX_FA_ptr_l	148	IDP_dMRI_ProbtrackX_MO_slf_l
72	IDP_dMRI_ProbtrackX_FA_ptr_r	149	IDP_dMRI_ProbtrackX_MO_slf_r
73	IDP_dMRI_ProbtrackX_FA_slf_l	150	IDP_dMRI_ProbtrackX_MO_str_l
74	IDP_dMRI_ProbtrackX_FA_slf_r	151	IDP_dMRI_ProbtrackX_MO_str_r
75	IDP_dMRI_ProbtrackX_FA_str_l	152	IDP_dMRI_ProbtrackX_MO_unc_l
76	IDP_dMRI_ProbtrackX_FA_str_r	153	IDP_dMRI_ProbtrackX_MO_unc_r
77	IDP_dMRI_ProbtrackX_FA_unc_l	154	IDP_dMRI_TBSS_MD_Middle_cerebellar_peduncle

bioRxiv preprint doi: https://doi.org/10.1101/2024.05.28.596120; this version posted June 1, 2024. The copyright holder for this preprint (which was not certified by peer review) is the author/funder, who has granted bioRxiv a license to display the preprint in perpetuity. It is made available under aCC-BY 4.0 International license.

Table S 10 Names of UK Biobank Imaging Derived Phenotypes (IDPs) related to White Matter-continued: 453. Supplement to **Figure 5**.

IDP #	IDP Name	IDP #	IDP Name
155	IDP_dMRI_TBSS_MD_Pontine_crossing_tract	232	IDP_dMRI_TBSS_ICVF_Body_of_corpus_callosum
156	IDP_dMRI_TBSS_MD_Genu_of_corpus_callosum	233	IDP_dMRI_TBSS_ICVF_Splenium_of_corpus_callosum
157	IDP_dMRI_TBSS_MD_Body_of_corpus_callosum	234	IDP_dMRI_TBSS_ICVF_Fornix
158	IDP_dMRI_TBSS_MD_Splenium_of_corpus_callosum	235	IDP_dMRI_TBSS_ICVF_Corticospinal_tract_R
159	IDP_dMRI_TBSS_MD_Fornix	236	IDP_dMRI_TBSS_ICVF_Corticospinal_tract_L
160	IDP_dMRI_TBSS_MD_Corticospinal_tract_R	237	IDP_dMRI_TBSS_ICVF_Medial_lemniscus_R
161	IDP_dMRI_TBSS_MD_Corticospinal_tract_L	238	IDP_dMRI_TBSS_ICVF_Medial_lemniscus_L
162	IDP_dMRI_TBSS_MD_Medial_lemniscus_R	239	IDP_dMRI_TBSS_ICVF_Inferior_cerebellar_peduncle_R
163	IDP_dMRI_TBSS_MD_Medial_lemniscus_L	240	IDP_dMRI_TBSS_ICVF_Inferior_cerebellar_peduncle_L
164	IDP_dMRI_TBSS_MD_Inferior_cerebellar_peduncle_R	241	IDP_dMRI_TBSS_ICVF_Superior_cerebellar_peduncle_R
165	IDP_dMRI_TBSS_MD_Inferior_cerebellar_peduncle_L	242	IDP_dMRI_TBSS_ICVF_Superior_cerebellar_peduncle_L
166	IDP_dMRI_TBSS_MD_Superior_cerebellar_peduncle_R	243	IDP_dMRI_TBSS_ICVF_Cerebral_peduncle_R
167	IDP_dMRI_TBSS_MD_Superior_cerebellar_peduncle_L	244	IDP_dMRI_TBSS_ICVF_Cerebral_peduncle_L
168	IDP_dMRI_TBSS_MD_Cerebral_peduncle_R	245	IDP_dMRI_TBSS_ICVF_Anterior_limb_of_internal_capsule_R
169	IDP_dMRI_TBSS_MD_Cerebral_peduncle_L	246	IDP_dMRI_TBSS_ICVF_Anterior_limb_of_internal_capsule_L
170	IDP_dMRI_TBSS_MD_Anterior_limb_of_internal_capsule_R	247	IDP_dMRI_TBSS_ICVF_Posterior_limb_of_internal_capsule_R
171	IDP_dMRI_TBSS_MD_Anterior_limb_of_internal_capsule_L	248	IDP_dMRI_TBSS_ICVF_Posterior_limb_of_internal_capsule_L
172	IDP_dMRI_TBSS_MD_Posterior_limb_of_internal_capsule_R	249	IDP_dMRI_TBSS_ICVF_Retrolenticular_part_of_internal_capsule_R
173	IDP_dMRI_TBSS_MD_Posterior_limb_of_internal_capsule_L	250	IDP_dMRI_TBSS_ICVF_Retrolenticular_part_of_internal_capsule_L
174	IDP_dMRI_TBSS_MD_Retrolenticular_part_of_internal_capsule_R	251	IDP_dMRI_TBSS_ICVF_Anterior_corona_radiata_R
175	IDP_dMRI_TBSS_MD_Retrolenticular_part_of_internal_capsule_L	252	IDP_dMRI_TBSS_ICVF_Anterior_corona_radiata_L
176	IDP_dMRI_TBSS_MD_Anterior_corona_radiata_R	253	IDP_dMRI_TBSS_ICVF_Superior_corona_radiata_R
177	IDP_dMRI_TBSS_MD_Anterior_corona_radiata_L	254	IDP_dMRI_TBSS_ICVF_Superior_corona_radiata_L
178	IDP_dMRI_TBSS_MD_Superior_corona_radiata_R	255	IDP_dMRI_TBSS_ICVF_Posterior_corona_radiata_R
179	IDP_dMRI_TBSS_MD_Superior_corona_radiata_L	256	IDP_dMRI_TBSS_ICVF_Posterior_corona_radiata_L
180	IDP_dMRI_TBSS_MD_Posterior_corona_radiata_R	257	IDP_dMRI_TBSS_ICVF_Posterior_thalamic_radiation_R
181	IDP_dMRI_TBSS_MD_Posterior_corona_radiata_L	258	IDP_dMRI_TBSS_ICVF_Posterior_thalamic_radiation_L
182	IDP_dMRI_TBSS_MD_Posterior_thalamic_radiation_R	259	IDP_dMRI_TBSS_ICVF_Sagittal_stratum_R
183	IDP_dMRI_TBSS_MD_Posterior_thalamic_radiation_L	260	IDP_dMRI_TBSS_ICVF_Sagittal_stratum_L
184	IDP_dMRI_TBSS_MD_Sagittal_stratum_R	261	IDP_dMRI_TBSS_ICVF_External_capsule_R
185	IDP_dMRI_TBSS_MD_Sagittal_stratum_L	262	IDP_dMRI_TBSS_ICVF_External_capsule_L
186	IDP_dMRI_TBSS_MD_External_capsule_R	263	IDP_dMRI_TBSS_ICVF_Cingulum_cingulate_gyrus_R
187	IDP_dMRI_TBSS_MD_External_capsule_L	264	IDP_dMRI_TBSS_ICVF_Cingulum_cingulate_gyrus_L
188	IDP_dMRI_TBSS_MD_Cingulum_cingulate_gyrus_R	265	IDP_dMRI_TBSS_ICVF_Cingulum_hippocampus_R
189	IDP_dMRI_TBSS_MD_Cingulum_cingulate_gyrus_L	266	IDP_dMRI_TBSS_ICVF_Cingulum_hippocampus_L
190	IDP_dMRI_TBSS_MD_Cingulum_hippocampus_R	267	IDP_dMRI_TBSS_ICVF_Fornix_cres+Stria_terminalis_R
191	IDP_dMRI_TBSS_MD_Cingulum_hippocampus_L	268	IDP_dMRI_TBSS_ICVF_Fornix_cres+Stria_terminalis_L
192	IDP_dMRI_TBSS_MD_Fornix_cres+Stria_terminalis_R	269	IDP_dMRI_TBSS_ICVF_Superior_longitudinal_fasciculus_R
193	IDP_dMRI_TBSS_MD_Fornix_cres+Stria_terminalis_L	270	IDP_dMRI_TBSS_ICVF_Superior_longitudinal_fasciculus_L
194	IDP_dMRI_TBSS_MD_Superior_longitudinal_fasciculus_R	271	IDP_dMRI_TBSS_ICVF_Superior_fronto-occipital_fasciculus_R
195	IDP_dMRI_TBSS_MD_Superior_longitudinal_fasciculus_L	272	IDP_dMRI_TBSS_ICVF_Superior_fronto-occipital_fasciculus_L
196	IDP_dMRI_TBSS_MD_Superior_fronto-occipital_fasciculus_R	273	IDP_dMRI_TBSS_ICVF_Uncinate_fasciculus_R
197	IDP_dMRI_TBSS_MD_Superior_fronto-occipital_fasciculus_L	274	IDP_dMRI_TBSS_ICVF_Uncinate_fasciculus_L
198	IDP_dMRI_TBSS_MD_Uncinate_fasciculus_R	275	IDP_dMRI_TBSS_ICVF_Tapetum_R
199	IDP_dMRI_TBSS_MD_Uncinate_fasciculus_L	276	IDP_dMRI_TBSS_ICVF_Tapetum_L
200	IDP_dMRI_TBSS_MD_Tapetum_R	277	IDP_dMRI_ProbtrackX_ICVF_ar_l
201	IDP_dMRI_TBSS_MD_Tapetum_L	278	IDP_dMRI_ProbtrackX_ICVF_ar_r
202	IDP_dMRI_ProbtrackX_MD_ar_l	279	IDP_dMRI_ProbtrackX_ICVF_atr_l
203	IDP_dMRI_ProbtrackX_MD_ar_r	280	IDP_dMRI_ProbtrackX_ICVF_atr_r
204	IDP_dMRI_ProbtrackX_MD_atr_l	281	IDP_dMRI_ProbtrackX_ICVF_cgc_l
205	IDP_dMRI_ProbtrackX_MD_atr_r	282	IDP_dMRI_ProbtrackX_ICVF_cgc_r
206	IDP_dMRI_ProbtrackX_MD_cgc_l	283	IDP_dMRI_ProbtrackX_ICVF_cgh_l
207	IDP_dMRI_ProbtrackX_MD_cgc_r	284	IDP_dMRI_ProbtrackX_ICVF_cgh_r
208	IDP_dMRI_ProbtrackX_MD_cgh_l	285	IDP_dMRI_ProbtrackX_ICVF_cst_l
209	IDP_dMRI_ProbtrackX_MD_cgh_r	286	IDP_dMRI_ProbtrackX_ICVF_cst_r
210	IDP_dMRI_ProbtrackX_MD_cst_l	287	IDP_dMRI_ProbtrackX_ICVF_fma
211	IDP_dMRI_ProbtrackX_MD_cst_r	288	IDP_dMRI_ProbtrackX_ICVF_fmi
212	IDP_dMRI_ProbtrackX_MD_fma	289	IDP_dMRI_ProbtrackX_ICVF_ifo_l
213	IDP_dMRI_ProbtrackX_MD_fmi	290	IDP_dMRI_ProbtrackX_ICVF_ifo_r
214	IDP_dMRI_ProbtrackX_MD_ifo_l	291	IDP_dMRI_ProbtrackX_ICVF_ilf_l
215	IDP_dMRI_ProbtrackX_MD_ifo_r	292	IDP_dMRI_ProbtrackX_ICVF_ilf_r
216	IDP_dMRI_ProbtrackX_MD_ilf_l	293	IDP_dMRI_ProbtrackX_ICVF_mcp
217	IDP_dMRI_ProbtrackX_MD_ilf_r	294	IDP_dMRI_ProbtrackX_ICVF_ml_l
218	IDP_dMRI_ProbtrackX_MD_mcp	295	IDP_dMRI_ProbtrackX_ICVF_ml_r
219	IDP_dMRI_ProbtrackX_MD_ml_l	296	IDP_dMRI_ProbtrackX_ICVF_ptr_l
220	IDP_dMRI_ProbtrackX_MD_ml_r	297	IDP_dMRI_ProbtrackX_ICVF_ptr_r
221	IDP_dMRI_ProbtrackX_MD_ptr_l	298	IDP_dMRI_ProbtrackX_ICVF_slf_l
222	IDP_dMRI_ProbtrackX_MD_ptr_r	299	IDP_dMRI_ProbtrackX_ICVF_slf_r
223	IDP_dMRI_ProbtrackX_MD_slf_l	300	IDP_dMRI_ProbtrackX_ICVF_str_l
224	IDP_dMRI_ProbtrackX_MD_slf_r	301	IDP_dMRI_ProbtrackX_ICVF_str_r
225	IDP_dMRI_ProbtrackX_MD_str_l	302	IDP_dMRI_ProbtrackX_ICVF_unc_l
226	IDP_dMRI_ProbtrackX_MD_str_r	303	IDP_dMRI_ProbtrackX_ICVF_unc_r
227	IDP_dMRI_ProbtrackX_MD_unc_l	304	IDP_dMRI_TBSS_OD_Middle_cerebellar_peduncle
228	IDP_dMRI_ProbtrackX_MD_unc_r	305	IDP_dMRI_TBSS_OD_Pontine_crossing_tract
229	IDP_dMRI_TBSS_ICVF_Middle_cerebellar_peduncle	306	IDP_dMRI_TBSS_OD_Genu_of_corpus_callosum
230	IDP_dMRI_TBSS_ICVF_Pontine_crossing_tract	307	IDP_dMRI_TBSS_OD_Body_of_corpus_callosum
231	IDP_dMRI_TBSS_ICVF_Genu_of_corpus_callosum	308	IDP_dMRI_TBSS_OD_Splenium_of_corpus_callosum

bioRxiv preprint doi: https://doi.org/10.1101/2024.05.28.596120; this version posted June 1, 2024. The copyright holder for this preprint (which was not certified by peer review) is the author/funder, who has granted bioRxiv a license to display the preprint in perpetuity. It is made available under aCC-BY 4.0 International license.

Table S 11 Names of UK Biobank Imaging Derived Phenotypes (IDPs) related to White Matter-continued: 453. Supplement to **Figure 5**.

IDP #	IDP Name	IDP #	IDP Name
309	IDP_dMRI_TBSS_OD_Fornix	386	IDP_dMRI_TBSS_ISOVF_Corticospinal_tract_L
310	IDP_dMRI_TBSS_OD_Corticospinal_tract_R	387	IDP_dMRI_TBSS_ISOVF_Medial_lemniscus_R
311	IDP_dMRI_TBSS_OD_Corticospinal_tract_L	388	IDP_dMRI_TBSS_ISOVF_Medial_lemniscus_L
312	IDP_dMRI_TBSS_OD_Medial_lemniscus_R	389	IDP_dMRI_TBSS_ISOVF_Inferior_cerebellar_peduncle_R
313	IDP_dMRI_TBSS_OD_Medial_lemniscus_L	390	IDP_dMRI_TBSS_ISOVF_Inferior_cerebellar_peduncle_L
314	IDP_dMRI_TBSS_OD_Inferior_cerebellar_peduncle_R	391	IDP_dMRI_TBSS_ISOVF_Superior_cerebellar_peduncle_R
315	IDP_dMRI_TBSS_OD_Inferior_cerebellar_peduncle_L	392	IDP_dMRI_TBSS_ISOVF_Superior_cerebellar_peduncle_L
316	IDP_dMRI_TBSS_OD_Superior_cerebellar_peduncle_R	393	IDP_dMRI_TBSS_ISOVF_Cerebral_peduncle_R
317	IDP_dMRI_TBSS_OD_Superior_cerebellar_peduncle_L	394	IDP_dMRI_TBSS_ISOVF_Cerebral_peduncle_L
318	IDP_dMRI_TBSS_OD_Cerebral_peduncle_R	395	IDP_dMRI_TBSS_ISOVF_Anterior_limb_of_internal_capsule_R
319	IDP_dMRI_TBSS_OD_Cerebral_peduncle_L	396	IDP_dMRI_TBSS_ISOVF_Anterior_limb_of_internal_capsule_L
320	IDP_dMRI_TBSS_OD_Anterior_limb_of_internal_capsule_R	397	IDP_dMRI_TBSS_ISOVF_Posterior_limb_of_internal_capsule_R
321	IDP_dMRI_TBSS_OD_Anterior_limb_of_internal_capsule_L	398	IDP_dMRI_TBSS_ISOVF_Posterior_limb_of_internal_capsule_L
322	IDP_dMRI_TBSS_OD_Posterior_limb_of_internal_capsule_R	399	IDP_dMRI_TBSS_ISOVF_Retrolenticular_part_of_internal_capsule_R
323	IDP_dMRI_TBSS_OD_Posterior_limb_of_internal_capsule_L	400	IDP_dMRI_TBSS_ISOVF_Retrolenticular_part_of_internal_capsule_L
324	IDP_dMRI_TBSS_OD_Retrolenticular_part_of_internal_capsule_R	401	IDP_dMRI_TBSS_ISOVF_Anterior_corona_radiata_R
325	IDP_dMRI_TBSS_OD_Retrolenticular_part_of_internal_capsule_L	402	IDP_dMRI_TBSS_ISOVF_Anterior_corona_radiata_L
326	IDP_dMRI_TBSS_OD_Anterior_corona_radiata_R	403	IDP_dMRI_TBSS_ISOVF_Superior_corona_radiata_R
327	IDP_dMRI_TBSS_OD_Anterior_corona_radiata_L	404	IDP_dMRI_TBSS_ISOVF_Superior_corona_radiata_L
328	IDP_dMRI_TBSS_OD_Superior_corona_radiata_R	405	IDP_dMRI_TBSS_ISOVF_Posterior_corona_radiata_R
329	IDP_dMRI_TBSS_OD_Superior_corona_radiata_L	406	IDP_dMRI_TBSS_ISOVF_Posterior_corona_radiata_L
330	IDP_dMRI_TBSS_OD_Posterior_corona_radiata_R	407	IDP_dMRI_TBSS_ISOVF_Posterior_thalamic_radiation_R
331	IDP_dMRI_TBSS_OD_Posterior_corona_radiata_L	408	IDP_dMRI_TBSS_ISOVF_Posterior_thalamic_radiation_L
332	IDP_dMRI_TBSS_OD_Posterior_thalamic_radiation_R	409	IDP_dMRI_TBSS_ISOVF_Sagittal_stratum_R
333	IDP_dMRI_TBSS_OD_Posterior_thalamic_radiation_L	410	IDP_dMRI_TBSS_ISOVF_Sagittal_stratum_L
334	IDP_dMRI_TBSS_OD_Sagittal_stratum_R	411	IDP_dMRI_TBSS_ISOVF_External_capsule_R
335	IDP_dMRI_TBSS_OD_Sagittal_stratum_L	412	IDP_dMRI_TBSS_ISOVF_External_capsule_L
336	IDP_dMRI_TBSS_OD_External_capsule_R	413	IDP_dMRI_TBSS_ISOVF_Cingulum_cingulate_gyrus_R
337	IDP_dMRI_TBSS_OD_External_capsule_L	414	IDP_dMRI_TBSS_ISOVF_Cingulum_cingulate_gyrus_L
338	IDP_dMRI_TBSS_OD_Cingulum_cingulate_gyrus_R	415	IDP_dMRI_TBSS_ISOVF_Cingulum_hippocampus_R
339	IDP_dMRI_TBSS_OD_Cingulum_cingulate_gyrus_L	416	IDP_dMRI_TBSS_ISOVF_Cingulum_hippocampus_L
340	IDP_dMRI_TBSS_OD_Cingulum_hippocampus_R	417	IDP_dMRI_TBSS_ISOVF_Fornix_cres+Stria_terminalis_R
341	IDP_dMRI_TBSS_OD_Cingulum_hippocampus_L	418	IDP_dMRI_TBSS_ISOVF_Fornix_cres+Stria_terminalis_L
342	IDP_dMRI_TBSS_OD_Fornix_cres+Stria_terminalis_R	419	IDP_dMRI_TBSS_ISOVF_Superior_longitudinal_fasciculus_R
343	IDP_dMRI_TBSS_OD_Fornix_cres+Stria_terminalis_L	420	IDP_dMRI_TBSS_ISOVF_Superior_longitudinal_fasciculus_L
344	IDP_dMRI_TBSS_OD_Superior_longitudinal_fasciculus_R	421	IDP_dMRI_TBSS_ISOVF_Superior_fronto-occipital_fasciculus_R
345	IDP_dMRI_TBSS_OD_Superior_longitudinal_fasciculus_L	422	IDP_dMRI_TBSS_ISOVF_Superior_fronto-occipital_fasciculus_L
346	IDP_dMRI_TBSS_OD_Superior_fronto-occipital_fasciculus_R	423	IDP_dMRI_TBSS_ISOVF_Uncinate_fasciculus_R
347	IDP_dMRI_TBSS_OD_Superior_fronto-occipital_fasciculus_L	424	IDP_dMRI_TBSS_ISOVF_Uncinate_fasciculus_L
348	IDP_dMRI_TBSS_OD_Uncinate_fasciculus_R	425	IDP_dMRI_TBSS_ISOVF_Tapetum_R
349	IDP_dMRI_TBSS_OD_Uncinate_fasciculus_L	426	IDP_dMRI_TBSS_ISOVF_Tapetum_L
350	IDP_dMRI_TBSS_OD_Tapetum_R	427	IDP_dMRI_ProbtrackX_ISOVF_ar_l
351	IDP_dMRI_TBSS_OD_Tapetum_L	428	IDP_dMRI_ProbtrackX_ISOVF_ar_r
352	IDP_dMRI_ProbtrackX_OD_ar_l	429	IDP_dMRI_ProbtrackX_ISOVF_atr_l
353	IDP_dMRI_ProbtrackX_OD_ar_r	430	IDP_dMRI_ProbtrackX_ISOVF_atr_r
354	IDP_dMRI_ProbtrackX_OD_atr_l	431	IDP_dMRI_ProbtrackX_ISOVF_cgc_l
355	IDP_dMRI_ProbtrackX_OD_atr_r	432	IDP_dMRI_ProbtrackX_ISOVF_cgc_r
356	IDP_dMRI_ProbtrackX_OD_cgc_l	433	IDP_dMRI_ProbtrackX_ISOVF_cgh_l
357	IDP_dMRI_ProbtrackX_OD_cgc_r	434	IDP_dMRI_ProbtrackX_ISOVF_cgh_r
358	IDP_dMRI_ProbtrackX_OD_cgh_l	435	IDP_dMRI_ProbtrackX_ISOVF_cst_l
359	IDP_dMRI_ProbtrackX_OD_cgh_r	436	IDP_dMRI_ProbtrackX_ISOVF_cst_r
360	IDP_dMRI_ProbtrackX_OD_cst_l	437	IDP_dMRI_ProbtrackX_ISOVF_fma
361	IDP_dMRI_ProbtrackX_OD_cst_r	438	IDP_dMRI_ProbtrackX_ISOVF_fmi
362	IDP_dMRI_ProbtrackX_OD_fma	439	IDP_dMRI_ProbtrackX_ISOVF_ifo_l
363	IDP_dMRI_ProbtrackX_OD_fmi	440	IDP_dMRI_ProbtrackX_ISOVF_ifo_r
364	IDP_dMRI_ProbtrackX_OD_ifo_l	441	IDP_dMRI_ProbtrackX_ISOVF_ilf_l
365	IDP_dMRI_ProbtrackX_OD_ifo_r	442	IDP_dMRI_ProbtrackX_ISOVF_ilf_r
366	IDP_dMRI_ProbtrackX_OD_ilf_l	443	IDP_dMRI_ProbtrackX_ISOVF_mcp
367	IDP_dMRI_ProbtrackX_OD_ilf_r	444	IDP_dMRI_ProbtrackX_ISOVF_ml_l
368	IDP_dMRI_ProbtrackX_OD_mcp	445	IDP_dMRI_ProbtrackX_ISOVF_ml_r
369	IDP_dMRI_ProbtrackX_OD_ml_l	446	IDP_dMRI_ProbtrackX_ISOVF_ptr_l
370	IDP_dMRI_ProbtrackX_OD_ml_r	447	IDP_dMRI_ProbtrackX_ISOVF_ptr_r
371	IDP_dMRI_ProbtrackX_OD_ptr_l	448	IDP_dMRI_ProbtrackX_ISOVF_slf_l
372	IDP_dMRI_ProbtrackX_OD_ptr_r	449	IDP_dMRI_ProbtrackX_ISOVF_slf_r
373	IDP_dMRI_ProbtrackX_OD_slf_l	450	IDP_dMRI_ProbtrackX_ISOVF_str_l
374	IDP_dMRI_ProbtrackX_OD_slf_r	451	IDP_dMRI_ProbtrackX_ISOVF_str_r
375	IDP_dMRI_ProbtrackX_OD_str_l	452	IDP_dMRI_ProbtrackX_ISOVF_unc_l
376	IDP_dMRI_ProbtrackX_OD_str_r	453	IDP_dMRI_ProbtrackX_ISOVF_unc_r
377	IDP_dMRI_ProbtrackX_OD_unc_l		
378	IDP_dMRI_ProbtrackX_OD_unc_r		
379	IDP_dMRI_TBSS_ISOVF_Middle_cerebellar_peduncle		
380	IDP_dMRI_TBSS_ISOVF_Pontine_crossing_tract		
381	IDP_dMRI_TBSS_ISOVF_Genu_of_corpus_callosum		
382	IDP_dMRI_TBSS_ISOVF_Body_of_corpus_callosum		
383	IDP_dMRI_TBSS_ISOVF_Splenium_of_corpus_callosum		
384	IDP_dMRI_TBSS_ISOVF_Fornix		
385	IDP_dMRI_TBSS_ISOVF_Corticospinal_tract_R		

bioRxiv preprint doi: https://doi.org/10.1101/2024.05.28.596120; this version posted June 1, 2024. The copyright holder for this preprint (which was not certified by peer review) is the author/funder, who has granted bioRxiv a license to display the preprint in perpetuity. It is made available under aCC-BY 4.0 International license.

Table S 12 Names of UK Biobank Non-Imaging Derived Phenotypes (nIDPs) related to Blood and Heart Health: 77. Supplement to **Figure 5**.

nIDP #	nIDP Name	nIDP #	nIDP Name
**1**	Pulse rate, automated reading (0.0),	**39**	Stroke volume during PWA (2.1),
**2**	Pulse rate, automated reading (0.1),	**40**	Mean arterial pressure during PWA (2.0),
**3**	Pulse rate, automated reading (2.0),	**41**	Mean arterial pressure during PWA (2.1),
**4**	Pulse rate, automated reading (2.1),	**42**	Ventricular rate (2.0),
**5**	Diastolic blood pressure, automated reading (0.0),	**43**	P duration (2.0),
**6**	Diastolic blood pressure, automated reading (0.1),	**44**	QRS duration (2.0),
**7**	Diastolic blood pressure, automated reading (2.0),	**45**	Systolic brachial blood pressure (2.0),
**8**	Diastolic blood pressure, automated reading (2.1),	**46**	Diastolic brachial blood pressure (2.0),
**9**	Systolic blood pressure, automated reading (0.0),	**47**	Cardiac index during PWA (2.0),
**10**	Systolic blood pressure, automated reading (0.1),	**48**	Cardiac index during PWA (2.1),
**11**	Systolic blood pressure, automated reading (2.0),	**49**	Pulse wave Arterial Stiffness index (0.0),
**12**	Systolic blood pressure, automated reading (2.1),	**50**	Pulse wave Arterial Stiffness index (2.0),
**13**	Pulse rate (0.0),	**51**	PQ interval (2.0),
**14**	Pulse rate (2.0),	**52**	QT interval (2.0),
**15**	Pulse wave reflection index (0.0),	**53**	QTC interval (2.0),
**16**	Pulse wave reflection index (2.0),	**54**	RR interval (2.0),
**17**	Pulse wave peak to peak time (0.0),	**55**	PP interval (2.0),
**18**	Pulse wave peak to peak time (2.0),	**56**	P axis (2.0),
**19**	Heart rate during PWA (2.0),	**57**	R axis (2.0),
**20**	Heart rate during PWA (2.1),	**58**	T axis (2.0),
**21**	Peripheral pulse pressure during PWA (2.0),	**59**	QRS num (2.0),
**22**	Central systolic blood pressure during PWA (2.0),	**60**	LV ejection fraction (2.0),
**23**	Central pulse pressure during PWA (2.0),	**61**	LV end diastolic volume (2.0),
**24**	Number of beats in waveform average for PWA (2.0),	**62**	LV end systolic volume (2.0),
**25**	Number of beats in waveform average for PWA (2.1),	**63**	LV stroke volume (2.0),
**26**	Central augmentation pressure during PWA (2.0),	**64**	Cardiac output (2.0),
**27**	Central augmentation pressure during PWA (2.1),	**65**	Average heart rate (2.0),
**28**	Augmentation index for PWA (2.0),	**66**	Minimum carotid IMT (intima-medial thickness) at 120 degrees (2.0),
**29**	Augmentation index for PWA (2.1),	**67**	Mean carotid IMT (intima-medial thickness) at 120 degrees (2.0),
**30**	Cardiac output during PWA (2.0),	**68**	Maximum carotid IMT (intima-medial thickness) at 120 degrees (2.0),
**31**	Cardiac output during PWA (2.1),	**69**	Minimum carotid IMT (intima-medial thickness) at 150 degrees (2.0),
**32**	End systolic pressure during PWA (2.0),	**70**	Mean carotid IMT (intima-medial thickness) at 150 degrees (2.0),
**33**	End systolic pressure during PWA (2.1),	**71**	Maximum carotid IMT (intima-medial thickness) at 150 degrees (2.0),
**34**	End systolic pressure index during PWA (2.0),	**72**	Minimum carotid IMT (intima-medial thickness) at 210 degrees (2.0),
**35**	End systolic pressure index during PWA (2.1),	**73**	Mean carotid IMT (intima-medial thickness) at 210 degrees (2.0),
**36**	Total peripheral resistance during PWA (2.0),	**74**	Maximum carotid IMT (intima-medial thickness) at 210 degrees (2.0),
**37**	Total peripheral resistance during PWA (2.1),	**75**	Minimum carotid IMT (intima-medial thickness) at 240 degrees (2.0),
**38**	Stroke volume during PWA (2.0),	**76**	Mean carotid IMT (intima-medial thickness) at 240 degrees (2.0),
		**77**	Maximum carotid IMT (intima-medial thickness) at 240 degrees (2.0)

bioRxiv preprint doi: https://doi.org/10.1101/2024.05.28.596120; this version posted June 1, 2024. The copyright holder for this preprint (which was not certified by peer review) is the author/funder, who has granted bioRxiv a license to display the preprint in perpetuity. It is made available under aCC-BY 4.0 International license.

Table S 13 Names of UK Biobank Non-Imaging Derived Phenotypes (nIDPs) related to Cognition: 68. Supplement to **Figure 5**.

nIDP #	nIDP Name
1	Number of incorrect matches in round (0.1)
2	Number of incorrect matches in round (0.2)
3	Number of incorrect matches in round (2.1)
4	Number of incorrect matches in round (2.2)
5	Number of incorrect matches in round (2.3)
6	Number of times snap-button pressed (0.0)
7	Number of times snap-button pressed (0.1)
8	Number of times snap-button pressed (0.2)
9	Number of times snap-button pressed (0.3)
10	Number of times snap-button pressed (0.4)
11	Number of times snap-button pressed (0.10)
12	Number of times snap-button pressed (0.11)
13	Number of times snap-button pressed (2.0)
14	Number of times snap-button pressed (2.1)
15	Number of times snap-button pressed (2.2)
16	Number of times snap-button pressed (2.3)
17	Number of times snap-button pressed (2.4)
18	Number of times snap-button pressed (2.5)
19	Number of times snap-button pressed (2.7)
20	Number of times snap-button pressed (2.10)
21	Number of times snap-button pressed (2.11)
22	Prospective memory result (0.0)
23	Prospective memory result (2.0)
24	FI8 : chained arithmetic (2.0)
25	FI9 : concept interpolation (2.0)
26	Duration to complete numeric path (trail #1) (2.0)
27	Mean time to correctly identify matches (2.0)
28	Number of incorrect matches in round (0.0)
29	Time elapsed (2.0)
30	Time elapsed (2.1)
31	Time elapsed (2.2)
32	Time elapsed (2.3)
33	Time elapsed (2.4)
34	Time elapsed (2.5)
35	Time elapsed (2.6)
36	Time elapsed (2.7)
37	Time elapsed (2.8)
38	Digits entered correctly (2.0)
39	Digits entered correctly (2.1)
40	Digits entered correctly (2.2)
41	Digits entered correctly (2.3)
42	Digits entered correctly (2.4)
43	Digits entered correctly (2.5)
44	Digits entered correctly (2.6)
45	Digits entered correctly (2.7)
46	Total errors traversing numeric path (trail #1) (2.0)
47	Mean time to correctly identify matches (0.0)
48	Fluid intelligence score (0.0)
49	Fluid intelligence score (2.0)
50	Duration to complete numeric path (trail #1) (0.0)
51	Duration to complete alphanumeric path (trail #2) (0.0)
52	Digits entered correctly (2.8)
53	Maximum digits remembered correctly (2.0)
54	Attempted fluid intelligence (FI) test. (2.0)
55	FI1 : numeric addition test (0.0)
56	FI1 : numeric addition test (2.0)
57	FI3 : word interpolation (0.0)
58	FI3 : word interpolation (2.0)
59	FI4 : positional arithmetic (0.0)
60	FI4 : positional arithmetic (2.0)
61	FI5 : family relationship calculation (0.0)
62	FI5 : family relationship calculation (2.0)
63	FI6 : conditional arithmetic (0.0)
64	FI6 : conditional arithmetic (2.0)
65	FI7 : synonym (0.0)
66	FI7 : synonym (2.0)
67	Duration to complete alphanumeric path (trail #2) (2.0)
68	Number of puzzles correctly solved (2.0)

bioRxiv preprint doi: https://doi.org/10.1101/2024.05.28.596120; this version posted June 1, 2024. The copyright holder for this preprint (which was not certified by peer review) is the author/funder, who has granted bioRxiv a license to display the preprint in perpetuity. It is made available under aCC-BY 4.0 International license.

Table S 14 Names of Human Connectome Project Non-Imaging Derived Phenotypes (nIDPs) used in CCA. Supplement to **Figure 5**.

nIDP #	nIDP Name	nIDP #	nIDP Name
1	PicVocab_Unadj	80	ASR_Witd_Pct
2	PicVocab_AgeAdj	81	IWRD_TOT
3	PMAT24_A_CR	82	PainInterf_Tscore
4	DDisc_AUC_200	83	MMSE_Score
5	THC	84	SSAGA_Alc_12_Frq_Drk
6	LifeSatisf_Unadj	85	Odor_Unadj
7	ListSort_AgeAdj	86	SSAGA_Alc_D4_Ab_Sx
8	ReadEng_Unadj	87	SSAGA_Mj_Use
9	SCPT_SPEC	88	ASR_Aggr_Raw
10	ReadEng_AgeAdj	89	SSAGA_Mj_Ab_Dep
11	ListSort_Unadj	90	DSM_Somp_Raw
12	DDisc_AUC_40K	91	FearSomat_Unadj
13	Avg_Weekday_Any_Tobacco_7days	92	SSAGA_Alc_12_Drinks_Per_Day
14	Num_Days_Used_Any_Tobacco_7days	93	Mars_Log_Score
15	Total_Any_Tobacco_7days	94	SelfEff_Unadj
16	PicSeq_AgeAdj	95	SCPT_SEN
17	FamHist_Fath_DrgAlc	96	NEOFAC_N
18	PicSeq_Unadj	97	SSAGA_Agoraphobia
19	Avg_Weekday_Cigarettes_7days	98	ASR_Intn_T
20	Avg_Weekend_Any_Tobacco_7days	99	AngHostil_Unadj
21	Total_Cigarettes_7days	100	Num_Days_Drank_7days
22	Dexterity_AgeAdj	101	SSAGA_Times_Used_Cocaine
23	Avg_Weekend_Cigarettes_7days	102	Loneliness_Unadj
24	Dexterity_Unadj	103	ASR_Intn_Raw
25	Times_Used_Any_Tobacco_Today	104	SSAGA_Alc_Hvy_Drinks_Per_Day
26	PSQI_Score	105	MeanPurp_Unadj
27	AngAggr_Unadj	106	DSM_Avoid_Pct
28	Taste_AgeAdj	107	NEOFAC_E
29	ASR_Rule_Raw	108	Total_Beer_Wine_Cooler_7days
30	Taste_Unadj	109	DSM_Avoid_Raw
31	ASR_Thot_Raw	110	Avg_Weekday_Wine_7days
32	EVA_Denom	111	Flanker_AgeAdj
33	SSAGA_TB_Still_Smoking	112	ASR_Anxd_Pct
34	FamHist_Fath_None	113	Avg_Weekend_Beer_Wine_Cooler_7days
35	ASR_Thot_Pct	114	SSAGA_Alc_D4_Ab_Dx
36	PercStress_Unadj	115	Total_Drinks_7days
37	ProcSpeed_AgeAdj	116	SSAGA_Alc_Hvy_Max_Drinks
38	ASR_Rule_Pct	117	FearAffect_Unadj
39	ProcSpeed_Unadj	118	Total_Wine_7days
40	DSM_Antis_Raw	119	Avg_Weekday_Drinks_7days
41	ER40_CR	120	ER40SAD
42	NEOFAC_A	121	Flanker_Unadj
43	ASR_Crit_Raw	122	ER40FEAR
44	VSPLOT_TC	123	Avg_Weekday_Beer_Wine_Cooler_7days
45	NEOFAC_O	124	SSAGA_Times_Used_Illicits
46	ER40ANG	125	Avg_Weekend_Drinks_7days
47	VSPLOT_OFF	126	SSAGA_Alc_D4_Dp_Sx
48	SSAGA_Times_Used_Stimulants	127	NEOFAC_C
49	ASR_Soma_Pct	128	Total_Hard_Liquor_7days
50	SSAGA_Mj_Times_Used	129	Correction
51	DSM_Antis_Pct	130	SSAGA_Alc_Hvy_Frq_5plus
52	CardSort_AgeAdj	131	DSM_Adh_Pct
53	ASR_Extn_Raw	132	ASR_Attn_Pct
54	ASR_Oth_Raw	133	VSPLOT_CRTE
55	ASR_Totp_T	134	SSAGA_Depressive_Ep
56	ASR_Extn_T	135	AngAffect_Unadj
57	ASR_Totp_Raw	136	SSAGA_PanicDisorder
58	EmotSupp_Unadj	137	Avg_Weekend_Hard_Liquor_7days
59	DSM_Anxi_Pct	138	FamHist_Moth_Dep
60	PercReject_Unadj	139	ASR_Anxd_Raw
61	ER40NOE	140	SSAGA_Times_Used_Opiates
62	DSM_Anxi_Raw	141	SSAGA_Times_Used_Sedatives
63	ASR_TAO_Sum	142	SSAGA_Alc_Hvy_Frq
64	SSAGA_TB_Smoking_History	143	SSAGA_Alc_12_Frq_5plus
65	CardSort_Unadj	144	Friendship_Unadj
66	PosAffect_Unadj	145	SSAGA_Depressive_Sx
67	SSAGA_ChildhoodConduct	146	ASR_Attn_Raw
68	Odor_AgeAdj	147	ASR_Intr_Raw
69	ASR_Witd_Raw	148	SSAGA_Alc_12_Frq
70	SSAGA_Alc_Hvy_Frq_Drk	149	FamHist_Fath_Dep
71	ASR_Soma_Raw	150	InstruSupp_Unadj
72	DSM_Depr_Pct	151	ASR_Intr_Pct
73	ASR_Aggr_Pct	152	SSAGA_Times_Used_Hallucinogens
74	SSAGA_Alc_12_Max_Drinks	153	Avg_Weekend_Wine_7days
75	DSM_Depr_Raw	154	FamHist_Moth_None
76	Mars_Final	155	Sadness_Unadj
77	PercHostil_Unadj	156	DSM_Hype_Raw
78	DSM_Somp_Pct	157	DSM_Adh_Raw
79	SSAGA_Alc_Age_1st_Use	158	DSM_Inat_Raw
